# PPARδ inhibition blocks the induction and function of tumor-induced IL-10^+^ regulatory B cells and enhances cancer immunotherapy

**DOI:** 10.1038/s41421-023-00568-6

**Published:** 2023-06-08

**Authors:** Chen Chen, Jianan Ma, Chenchen Pi, Wei Huang, Tao Zhang, Cong Fu, Wentao Liu, Yong-Guang Yang

**Affiliations:** 1 Key Laboratory of Organ Regeneration and Transplantation of the Ministry of Education, The First Hospital of Jilin University, Changchun, Jilin, China; 2 National-Local Joint Engineering Laboratory of Animal Models for Human Diseases, Jilin University, Changchun, Jilin, China; 3 Centre of Oncology, The First Hospital of Jilin University, Changchun, Jilin, China; 4 International Center of Future Science, Jilin University, Changchun, Jilin, China

**Keywords:** Tumour immunology, Cancer microenvironment

## Abstract

IL-10^+^ regulatory B cells (Bregs) play a significant role in cancer immunotherapy and their presence is an indicator of negative outcome. We found that PPARδ is significantly upregulated in tumor-induced IL-10^+^ Bregs with a phenotype of CD19^+^CD24^hi^IgD^lo/−^CD38^lo^ or CD19^+^CD24^hi^IgD^lo/−^CD38^hi^ in both mice and humans, and the level of PPARδ expression was correlated with their potential to produce IL-10 and to inhibit T cell activation. Genetic inactivation of PPARδ in B cells impaired the development and function of IL-10^+^ B cells, and treatment with PPARδ inhibitor diminished the induction of IL-10^+^ Bregs by tumor and CD40 engagement. Importantly, immunotherapy with anti-CD40 or anti-PD1 antibody achieved a markedly improved outcome in tumor-bearing mice with PPARδ deficiency in B cells or treated with PPARδ inhibitor. This study shows that PPARδ is required for the development and function of IL-10^+^ Bregs, providing a new and effective target for selectively blocking Bregs and improving antitumor immunotherapy.

## Introduction

Cancer immunotherapy has made revolutionary progress and produced remarkable results in recent years. It can be broadly classified into two types according to mechanisms of action. One is to resume or normalize pre-existing antitumor immune responses by the use of immune checkpoint (e.g., PD-1/PD-L1) blockades^1,2^. Although this type of treatment is effective both experimentally and clinically, it works only for “hot” tumors that represent only a small portion of cancer patients^1^. The other approach is to elicit and activate antitumor immune responses by, e.g., agonists of dendritic cell (DC) and T cell activation, such as CD40 agonist mAbs^3^, which has the potential to covert cold tumors to hot^4^.

Emerging evidence demonstrates the presence of tumor-associated immunosuppressive B cells or regulatory B cells (Bregs) that limit the efficacy of cancer immunotherapy^5–7^. However, B cells were also reported to elicit antitumor immunity^8–12^. In agreement with these conflicting findings, B cell depletion was also found ineffective^13,14^ or detrimental^15^ in other studies. These findings implicate that tumor-associated B cells are a highly heterogeneous population, consisting of both immuno-stimulatory B cells and immunosuppressive Bregs. Thus, understanding the insights into tumor-induced Bregs and developing strategies for specifically inhibiting Bregs are of paramount importance. Bregs are mainly defined by their effector molecules. Unlike regulatory T cells (Tregs), for which the generation, phenotypes and function were well identified, Bregs remain highly elusive for their differentiation and phenotypes, making specific Breg inactivation highly challenging^7,12,16^.

In the present study, we discovered that peroxisome proliferator-activated receptor delta (PPARδ) is highly expressed in IL-10-producing Bregs and serves as a key regulator controlling their development and function in tumor-bearing mice and cancer patients. Furthermore, combination therapy with PPARδ inhibitor and anti-CD40 mAb or anti-PD1 achieved synergistically improved antitumor responses.

## Results

### PPARδ inhibitor enhances the therapeutic effect of agonistic anti-CD40 mAb

**Fig. 1:**
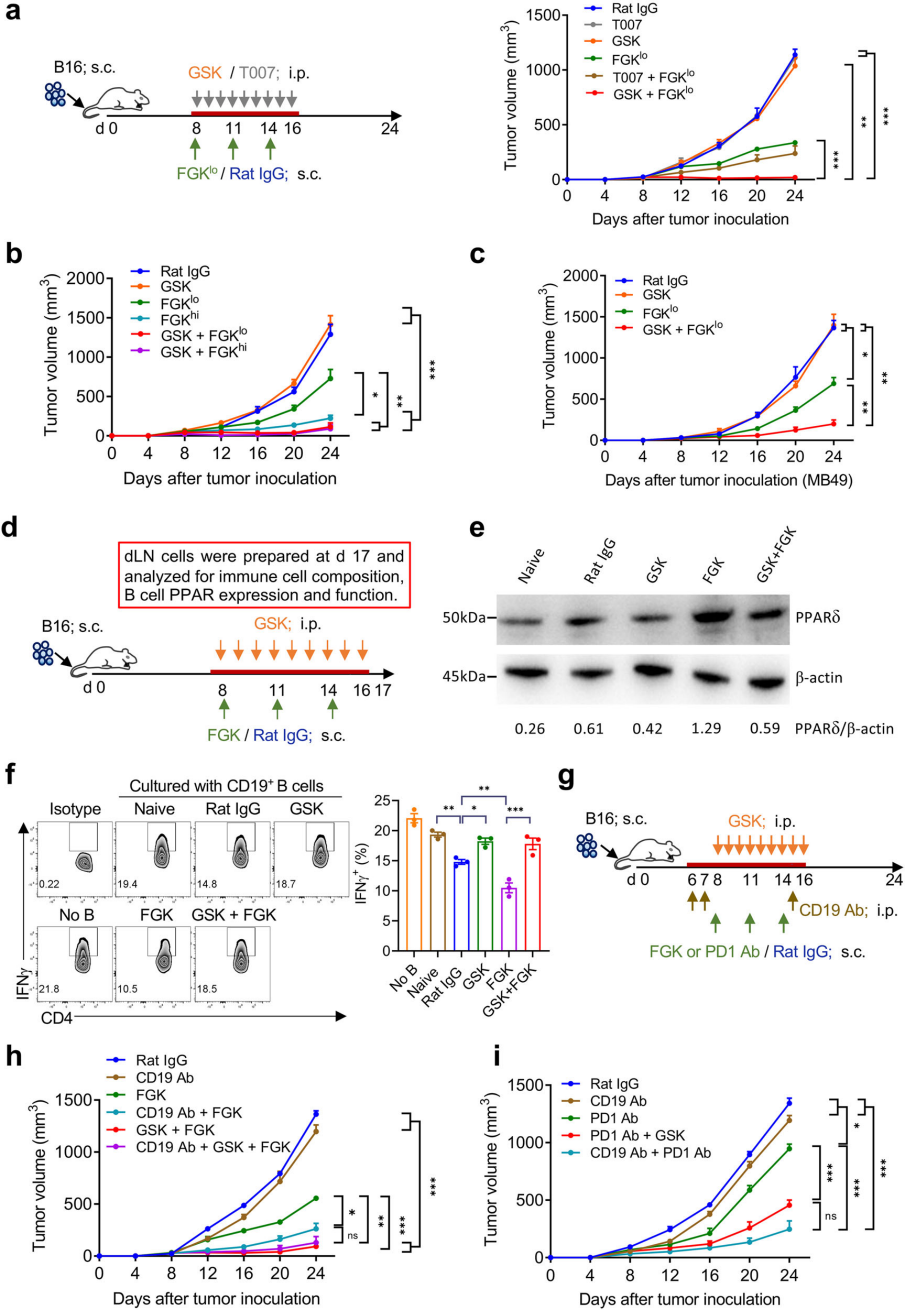
PPARδ inhibitor induces improved antitumor effects of anti-CD40 agonist mAb or anti-PD1 mAb through blocking tumor-induced expansion of immunosuppressive B cells. **a** C57BL/6 mice were inoculated s.c. with 7 × 10^5^ B16 cells and treated 8 days later with FGK or Rat IgG (peritumorally s.c.; 25 μg per day at day 8, 11 and 14; FGK^lo^), T007, GSK, FGK plus T007, or FGK plus GSK. T007 (45 nmol) or GSK (300 nmol) was administrated i.p. daily from day 8 to 16. Shown are schematic diagram of experiment setup (left; *n* = 5–6) and data of tumor growth (right). **b** C57BL/6 mice (*n* = 8–9) were inoculated B16 cells, followed 8 days later by peritumoral injection of low-dose (25 µg × 3; FGK^lo^) or high-dose (40 µg × 5; FGK^hi^) FGK or Rat IgG (40 µg × 5). GSK was administrated i.p. daily from day 8 to 16 or 22 (GSK alone group was given from day 8 to 22). Shown are data of tumor growth. **c** Tumor growth in C57BL/6 mice (*n* = 5) that were inoculated s.c. with 7 × 10^5^ MB49 cells, and treated with FGK or Rat IgG, GSK, or FGK plus GSK as described in (**a**). **d**–**f** C57BL/6 mice were inoculated s.c. with 7 × 10^5^ B16 cells and treated 8 days later by FGK or Rat IgG (peritumorally s.c.; 25 μg per day at days 8, 11 and 14), or GSK (300 nmol; daily from day 8 to day 16), or FGK plus GSK. dLNs (inguinal LNs) were harvested at day 17 and analyzed for PPARδ expression in B cells and B cell function. Shown are schematic diagram of experimental setup (**d**), PPARδ expression in purified dLN B cells analyzed by western blot (*n* = 3/group) (**e**), and representative FACS profiles (left) and percentages (right) of IFNγ^+^ cells in CD4^+^CD25^−^ T cells (**f**). **g**–**i** C57BL/6 mice (*n* = 5–7) were inoculated with B16 cells, and treated with Rat IgG, FGK (s.c.), PD-1 Ab (i.p.; 100 µg/day at days 8, 11 and 14), GSK, or in combinations; some groups of mice were depleted of B cells by anti-CD19 mAbs (i.p.; 250 µg per day at days 6, 7 and 15). **g** Schematic diagram of experimental setup. Tumor growth for mice receiving immunotherapy with FGK (**h**) or anti-PD-1Ab (**i**). Data are means ± SEM, or representative staining profiles or images. *P* values were determined by two-way ANOVA with Tukey’s multiple comparisons test (**a**–**c** and **h**, **i**) or one-way ANOVA with Tukey’s multiple comparisons test (**f**). Shown are data from a representative of three (**a**–**f**) or two (**h**, **i**) independent experiments.

Genetic sensors of fatty acids, PPARγ and PPARδ are required for maturation of alternatively activated macrophages (M2) in adipose tissues and disruption of PPARγ or PPARδ results in macrophage polarization toward M1 state^17–20^. Reversing macrophage polarization from the immunosuppressive M2 to M1 state has been proven to improve cancer immunotherapy^21,22^. We therefore determined whether agonist anti-CD40 mAb (FGK45.5 or FGK) in combination with PPARγ (T0070907 or T007) or PPARδ (GSK3787 or GSK) inhibitor may achieve efficient antitumor responses in mice with established B16 melanoma (Fig. 1a, left). In this model, although anti-CD40 mAb (given peritumorally to reduce its toxicity)^23^ induced a dose-dependent antitumor response (Supplementary Fig. S1a), the treatment also resulted in liver toxicity in a dose-dependent manner as shown by tissue damage with inflammatory cellular infiltrations (Supplementary Fig. S1b) and increased serum levels of alanine aminotransferase (ALT) (Supplementary Fig. S1c). Compared to B16 melanoma-bearing mice treated with FGK (75 μg divided into three injections with a 3-day interval) alone that showed significant improvement relative to Rat IgG controls, profoundly improved antitumor responses were seen in mice receiving FGK in combination with GSK, but not T007 (Fig. 1a, right). Although FGK at a higher dose (200 μg divided into five injections with a 3-day interval) induced a significantly stronger antitumor activity when used alone, no difference was detected between mice receiving high vs. low dose of FGK when used in combination with GSK (Fig. 1b and Supplementary Fig. S1d). GSK did not induce detectable hepatotoxicity when used alone (Supplementary Fig. S1e), or aggravate hepatotoxicity induced by FGK (Supplementary Fig. S1c, e, f). A similar observation was made in a mouse bladder carcinoma (MB49) model, in which combination therapy with FGK and GSK also induced a significantly improved antitumor effect compared to FGK alone (Fig. 1c). The enhanced antitumor effect by combination therapy was associated with significantly increased CD3^+^, in particular CD3^+^CD8^+^ tumor-infiltrating lymphocytes (TILs) (Supplementary Fig. S2a, b). While CD8 T cells from different groups of tumor-bearing mice expressed comparable levels of perforin, Granzyme B and CD107a, PD1 expression appeared to be reduced in mice treated with FGK (Supplementary Fig. S2c), which is consistent with previous findings^24,25^. Immunohistochemistry (IHC) results revealed that combination treatment not only increased the density, but also infiltration depth of CD8^+^ TILs inside the tumor (Supplementary Fig. S2d).

Previous studies have shown that the antitumor effect of CD40 agonist antibody is T cell-dependent^23,26^ and induction of immune memory is essential to prevent cancer recurrence^27,28^. Thus, we assessed antitumor memory immune responses in tumor-free mice that were initially inoculated (subcutaneously, s.c.) with B16 melanoma and treated with low-dose FGK (75 µg) plus GSK or with high-dose FGK (200 µg) alone (Supplementary Fig. S2e, top). These mice were re-challenged with intravenous (i.v.) injection of 7 × 10^5^ B16 cells 90 days after initial tumor inoculation, and age-matched naïve mice without previous tumor challenge were used as the control (Supplementary Fig. S2e, top). Compared to naïve control mice that all died by 31 days, mice that survived first tumor challenge in both combination therapy and FGK alone groups showed significantly improved long-term survival (Supplementary Fig. S2e, bottom), indicating the presence of antitumor memory immune responses in these mice. It appeared that the combination therapy group had a higher long-term survival rate (86%) than the FGK alone group (50%), indicative of a stronger memory response in the former group. Together, these results indicate that PPARδ inhibitor may synergistically enhance the antitumor effect of anti-CD40 mAb without aggravating its toxicity, offering a potential approach to improve immunotherapy with anti-CD40 mAb at tolerable doses.

### PPARδ inhibitor acts synergistically with immunotherapy to enhance antitumor immunity by blocking the immunosuppressive function of B cells

Because both PPARγ and PPARδ inhibitors were reported to inhibit tumor-induced M2 macrophage polarization^17–20^, the different effects of PPARγ vs. PPARδ inhibitors to enhance immunotherapy with anti-CD40 mAb suggest that tumor-associated macrophages (TAMs) were not the targeting cells as we initially hypothesized. In support of this possibility, we found that there was no significant difference in the frequency and quantification of PPARγ^+^ cells or the level of PPARγ expression among CD11b^+^F4/80^+^ monocytes/macrophages (Supplementary Fig. S3a, b) between untreated and T007-injected tumor-bearing mice, and that levels of PPARδ expression in macrophages were comparable between naïve and tumor-bearing mice, and not affected by treatment with FGK or GSK (Supplementary Fig. S3c). However, PPARδ expression was unexpectedly found to be significantly upregulated in B cells from draining lymph nodes (dLNs) of tumor-bearing mice (Fig. 1d, e), while PPARγ expression was undetectable in these B cells (data not shown). Compared to naïve mouse B cells, increased PPARδ expression was observed in B cells from tumor-bearing mice treated with Rat IgG but not with GSK, and this tumor-induced PPARδ upregulation in B cells was further elevated in mice treated with FGK but not with FGK plus GSK (Fig. 1e). Together, these data suggest that the effect of PPARδ inhibitor to enhance the antitumor activity of CD40 activation was likely mediated by targeting B cells rather than TAMs.

These results prompted us to analyze B cells in dLNs where T cell priming to tumor antigens occurs. To determine the function of B cells in dLNs of tumor-bearing mice, we evaluated their ability to suppress IFNγ production of T cells in response to anti-CD3/CD28 stimulation in vitro. Compared to B cells from naïve mice that had a moderate inhibitory effect, significantly stronger inhibition of CD4 T cell IFNγ production was induced by B cells from tumor-bearing mice (Rat IgG-treated controls), which was further enhanced by FGK treatment (Fig. 1f), indicating that tumor formation and CD40 agonist may augment immunosuppressive B cells. However, the potential of tumor and CD40 agonist to promote the immunosuppressive activity of B cells was markedly suppressed GSK (Fig. 1f). Furthermore, dLN B cells from tumor-bearing mice treated with GSK alone or along with FGK also showed a significantly reduced ability to inhibit IFNγ production by CD8 T cells (regardless of whether CD8 T cells were cultured with or without CD4^+^CD25^−^ T cells; Supplementary Fig. S4a–c), and the production of perforin and granzyme B by CD8 T cells (Supplementary Fig. S4d–f), compared to those from tumor-bearing mice treated with Rat IgG or FGK. It appeared that a significant increase in the proportion of IFNγ-producing CD8 T cells was found when CD8 T cells were cultured with CD4^+^CD25^−^ T cells compared to those cultured alone (Supplementary Fig. S4a–c). In line with this observation, B cell depletion significantly enhanced antitumor effects in mice treated with FGK alone, but not in those receiving combination therapy with FGK and GSK (Fig. 1g, h). Similar to synergism with FGK, PPARδ inhibitor or B cell depletion also acted synergistically to promote the antitumor effect of anti-PD-1 antibodies (Fig. 1i). Together, these results suggest that PPARδ plays a critical role in the development of tumor-induced immunosuppressive B cells (i.e., Bregs) that inhibit the antitumor effect of immunotherapy.

### PPARδ is highly expressed by IL-10^+^ Bregs with a surface phenotype of CD24^hi^IgD^lo/−^CD38^lo^ or CD24^hi^IgD^lo/−^CD38^hi^

**Fig. 2:**
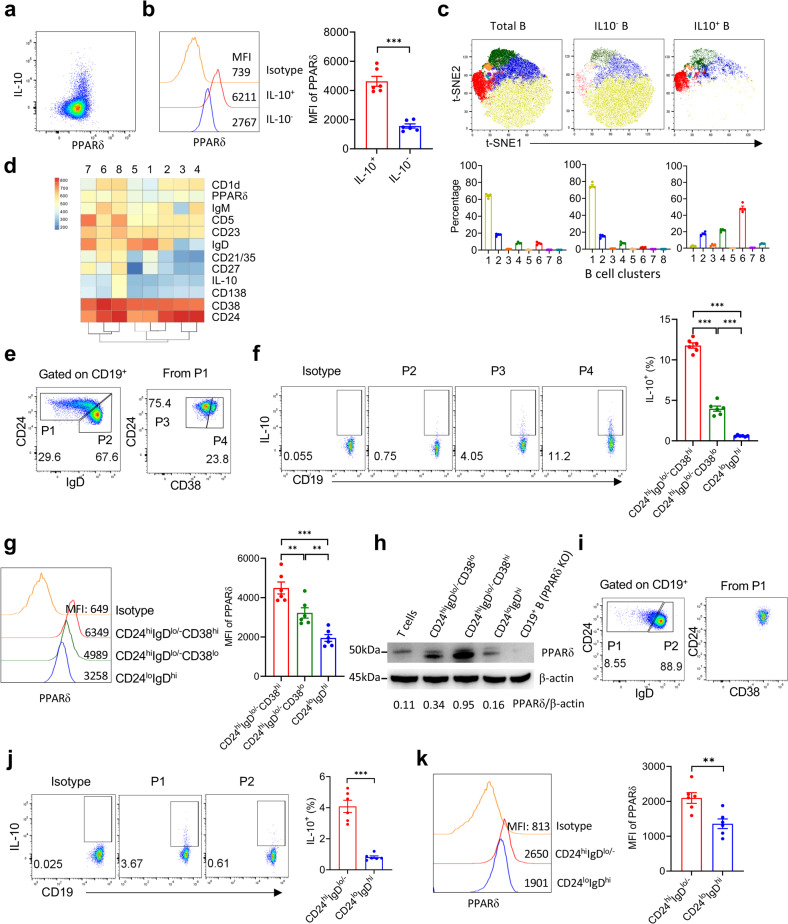
PPARδ is highly expressed by IL-10^+^ Bregs with a surface phenotype of CD24^hi^IgD^lo/−^CD38^lo^ or CD24^hi^IgD^lo/−^CD38^hi^. **a** Representative FACS profiles showing IL-10 and PPARδ expression in naïve mouse splenic CD19^+^ B cells. **b** Representative FACS profiles (left) and levels (MFI) of PPARδ expression (right) in naïve mouse splenic CD19^+^IL-10^−^ and CD19^+^IL-10^+^ B cells. **c**, **d** Naïve mouse splenic B cells were analyzed by multi-color flow cytometry and clustered by the FlowSOM algorithm. **c** t-SNE plots (top panel) and percentages (bottom panel) of the identified B cell clusters in total, IL-10^−^ and IL-10^+^ B cells. **d** Heat map showing relative expression levels of the indicated markers in each CD19^+^ B cell clusters. **e**–**h** IL-10 secretion and PPARδ expression in naïve mouse splenic CD19^+^CD24^hi^IgD^lo/−^CD38^lo^ Bregs, CD19^+^CD24^hi^IgD^lo/−^CD38^hi^ Bregs and CD19^+^CD24^lo^IgD^hi^ mature B cells. **e** Gating strategy. **f** Representative profiles showing IL-10 expression (left) and percentages (*n* = 6) of IL-10^+^ cells (right). **g** Representative FACS profiles (left) and levels (MFI) of PPARδ expression (right). **h** PPARδ expression in each sorted B cell subsets measured by western blot assay (WT mouse naïve T cells and PPARδ KO mouse naïve B cells were used as controls). **i**–**k** IL-10 secretion and PPARδ expression in naïve mouse LN CD19^+^CD24^hi^IgD^lo/−^CD38^lo^ Bregs and CD19^+^CD24^lo^IgD^hi^ mature B cells. **i** Gating strategy. **j** Representative profiles showing IL-10 expression (left) and percentages (*n* = 6) of IL-10^+^ cells (right). **k** Representative FACS profiles (left) and levels (MFI) of PPARδ expression (right). Data presented are means ± SEM, representative staining profiles or western blot images. *P* values were determined by unpaired two-tailed Student’s *t* test (**b**, **j** and **k**) or one-way ANOVA with Tukey’s multiple comparisons test (**f**, **g**). Shown are data from a representative of three (**a**–**g**, **i**–**k**) or two (**h**) independent experiments.

Bregs are mainly defined by their effector molecules. Although multiple effector molecules have been reported, IL-10 is considered the main mediator of tumor-associated Breg function^5,29–32^. Therefore, we compared PPARδ expression between splenic IL-10^+^ Bregs and IL-10^−^ B cells, and found that PPARδ was expressed significantly higher in the former than the latter population (Fig. 2a, b). To reveal IL-10^+^ Breg surface phenotypes, multi-color flow cytometry was performed, and B cells were clustered and visualized in 2-dimension using t-distributed stochastic neighbor embedding (t-SNE). We found that the proportion distribution of the identified eight B cell clusters in IL-10^+^ Bregs was distinctive from total B cells and IL-10^−^ B cells (the latter two groups were comparable) (Fig. 2c, d). Furthermore, the major clusters in IL-10^+^ Bregs (clusters 2, 4 and 6) and the clusters that were clearly detected in IL-10^+^ Bregs but almost undetectable in IL-10^−^ B cells (clusters 3 and 8) were all CD24^hi^, IgD^lo/−^ and CD38^lo/hi^ (Fig. 2c, d). While both CD24^hi^IgD^lo/–^CD38^lo^ and CD24^hi^IgD^lo/−^CD38^hi^ splenic B cells consisted of significantly higher proportions of IL-10^+^ cells than the rest CD24^lo^IgD^hi^ mature B cells, the latter subset was further enriched for IL-10^+^ cells (Fig. 2e, f). Furthermore, flow cytometry and western blot analysis revealed that the frequency of IL-10^+^ B cells positively correlated with the level of PPARδ expression (Fig. 2g, h). CD24^hi^IgD^lo/–^CD38^lo^ and CD24^hi^IgD^lo/–^CD38^hi^ B cells were also detected in peripheral blood (Supplementary Fig. S5a), and these cells also expressed higher levels of PPARδ and IL-10 than CD24^lo^IgD^hi^ B cells (Supplementary Fig. S5b, c). Unlike splenic and peripheral blood B cells, CD24^hi^IgD^lo/–^ B cells in lymph nodes were mainly CD38^lo^ (i.e., CD24^hi^IgD^lo/–^CD38^lo^) (Fig. 2i), which also consisted of a significantly higher frequency of IL-10^+^ cells (Fig. 2j) and expressed a higher level of PPARδ (Fig. 2k) than mature (CD24^lo^IgD^hi^) B cells. These results indicate that PPARδ is significantly upregulated in IL-10^+^ Bregs that are phenotypically characterized as CD24^hi^IgD^lo/–^CD38^lo^ or CD24^hi^IgD^lo/–^CD38^hi^, and the level of PPARδ expression positively correlates with their potential to produce IL-10.

### PPARδ inhibitor inhibits the development and function of IL-10^+^ Bregs induced by tumor and agonistic CD40 antibody

**Fig. 3:**
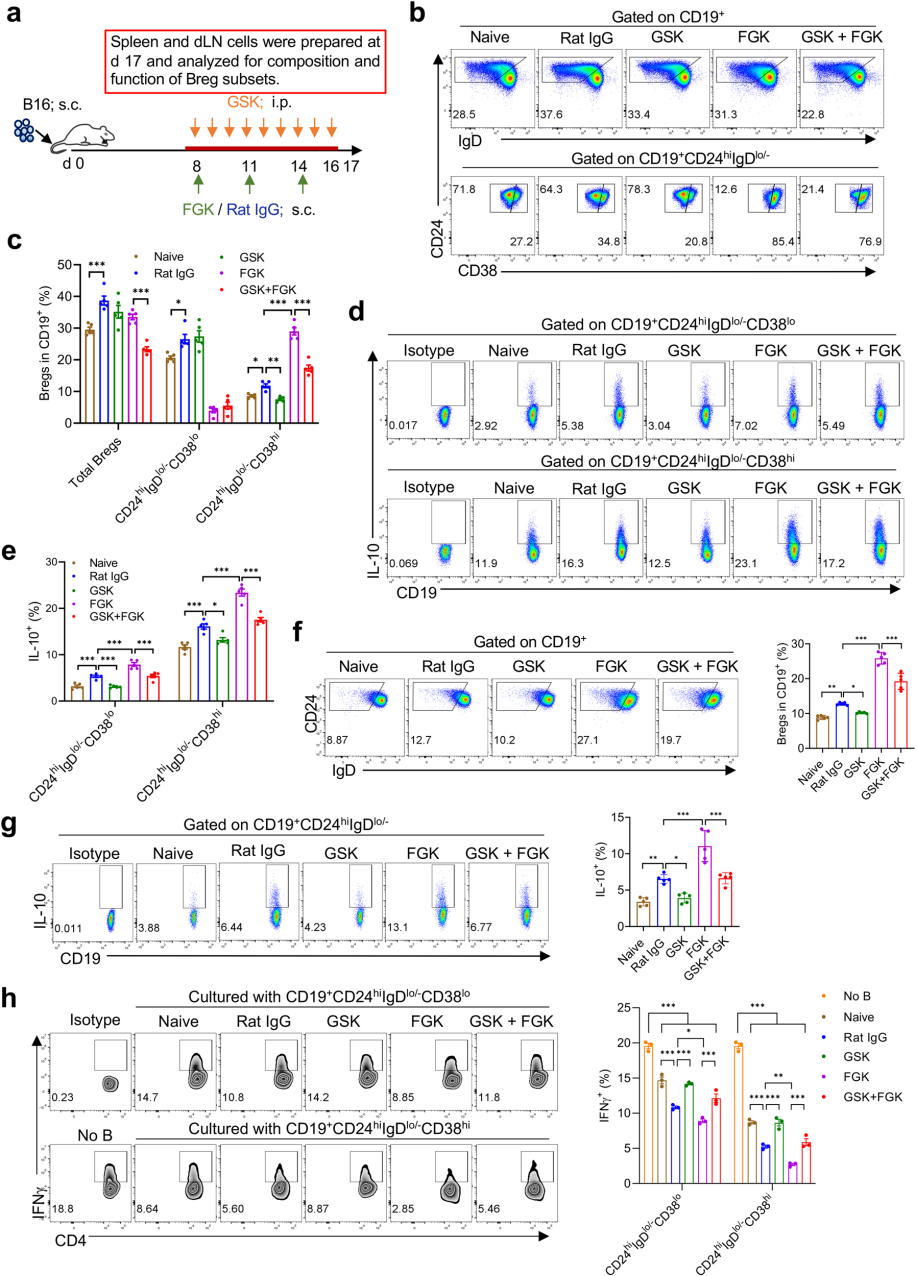
GSK blocks the induction and immunosuppressive function of tumor-associated Bregs. **a** Schematic diagram of experimental setup. **b** Representative profiles showing CD24, IgD and CD38 expression on gated CD19^+^ cells. **c** Percentages of total, CD19^+^CD24^hi^IgD^lo/−^CD38^lo^, and CD19^+^CD24^hi^IgD^lo/−^CD38^hi^ Bregs in splenic B cells of naive or tumor-bearing mice receiving the indicated treatment. Representative profiles showing IL-10 staining (**d**) and percentages of IL-10^+^ cells (**e**) in splenic CD19^+^CD24^hi^IgD^lo/−^CD38^lo^ and CD19^+^CD24^hi^IgD^lo/−^CD38^hi^ Bregs from the indicated groups. **f** Representative profiles showing CD24 and IgD expression on gated CD19^+^ cells (left), and percentages of CD19^+^CD24^hi^IgD^lo/−^ B cells in dLN B cells (right) of naive or tumor-bearing mice from different treatment groups. **g** Representative profiles showing IL-10 staining (left) and percentages of IL-10^+^ cells (right) in dLN CD19^+^CD24^hi^IgD^lo/−^ Bregs from the indicated groups. **h** CD19^+^CD24^hi^IgD^lo/−^CD38^lo^, CD19^+^CD24^hi^IgD^lo/−^CD38^hi^ and CD19^+^CD24^lo^IgD^hi^ B cells were sorted from the indicated groups and examined for the ability to inhibit IFNγ production of CD4^+^CD25^−^ T cells in response to CD3/CD28 stimulation. Shown are representative FACS profiles (left) and percentages (right) of IFNγ^+^ cells in CD4^+^ T cells. All data are means ± SEM, and *P* values were determined by one-way ANOVA with Tukey’s multiple comparisons test (**c**, **e**–**h**). Shown are data from a representative of three (**b**–**e**, **h**) or two (**f**, **g**) independent experiments.

We next characterized CD24^hi^IgD^lo/–^CD38^lo^ and CD24^hi^IgD^lo/–^CD38^hi^ IL-10^+^ Bregs in tumor-bearing mice and their responses to FGK or GSK. Tumor-bearing mice were treated with FGK or GSK alone or in combination, then spleen and dLN cells were prepared 3 days after the last FGK treatment or 1 day after the last GSK injection and analyzed for B cell phenotypes (Fig. 3a). We found that, compared to naïve mice, a significant increase in splenic CD24^hi^IgD^lo/–^CD38^lo^ and CD24^hi^IgD^lo/–^CD38^hi^ Bregs, in particular the latter subset, was seen in tumor-bearing mice treated with Rat IgG, but not in those treated with GSK (Fig. 3b, c). Although total Bregs were comparable between tumor-bearing mice treated with Rat IgG vs. FGK, FGK treatment resulted in a significant increase in the frequency of CD24^hi^IgD^lo/−^CD38^hi^ Bregs, and the magnitude of FGK-induced increase in CD24^hi^IgD^lo/−^CD38^hi^ Bregs was significantly reduced by GSK (Fig. 3b, c). In line with the increase in numbers, Ki-67 staining revealed that CD24^hi^IgD^lo/–^CD38^lo^ and CD24^hi^IgD^lo/−^CD38^hi^ Bregs in tumor-bearing mice showed greater proliferation than those in naïve mice, which was further increased by FGK and inhibited by GSK (Supplementary Fig. S6a). However, in contrast to FGK-induced increase in Breg frequencies (Fig. 3b, c), CD24^hi^IgD^lo/−^CD38^lo^ Bregs proliferated more robustly than CD24^hi^IgD^lo/−^CD38^hi^ Bregs in tumor-bearing mice treated with FGK (Supplementary Fig. S6a), suggesting that FGK may promote transition of CD24^hi^IgD^lo/−^CD38^lo^ Bregs to CD24^hi^IgD^lo/−^CD38^hi^ Bregs. IL-10 secretion by both Breg subsets was also increased in tumor-bearing mice treated with Rat IgG, but not in those-treated with GSK, compared to those of naïve mice (Fig. 3d, e). Furthermore, IL-10 secretion by these Bregs was markedly elevated in tumor-bearing mice treated with FGK, but to significantly less magnitude in those treated with FGK plus GSK (Fig. 3d, e). Similar results were obtained for dLN Bregs. Compared to naïve mice, the frequency (Fig. 3f), proliferation (Supplementary Fig. S6b) and IL-10 secretion (Fig. 3g) of CD24^hi^IgD^lo/−^CD38^lo^ Bregs in dLNs (similar to naïve mouse LNs, dLNs from tumor-bearing mice also lacked CD24^hi^IgD^lo/−^CD38^hi^ Bregs; Supplementary Fig. S7) were significantly elevated in tumor-bearing mice treated with Rat IgG, but not those treated with GSK. Moreover, although increased frequency (Fig. 3f), proliferation (Supplementary Fig. S6b) and IL-10 secretion (Fig. 3g) of CD24^hi^IgD^lo/−^CD38^lo^ Bregs in dLNs were detected in tumor-bearing mice treated with FGK compared to the controls treated with Rat IgG, the levels were significantly lower in mice treated with FGK plus GSK.

To further determine the immunosuppressive activity of CD24^hi^IgD^lo/−^CD38^lo^ and CD24^hi^IgD^lo/−^CD38^hi^ Bregs, these cells were isolated from naïve or tumor-bearing mice, and assessed for their ability to suppress T cell activation by measuring IFNγ production following anti-CD3/CD28 stimulation. Although both CD24^hi^IgD^lo/−^CD38^lo^ and CD24^hi^IgD^lo/−^CD38^hi^ Bregs were capable of inhibiting IFNγ production by T cells, the latter was superior to the former (Fig. 3h and Supplementary Fig. S8). Furthermore, both Breg subsets from tumor-bearing mice treated with Rat IgG, but not those treated with GSK, showed stronger inhibition on IFNγ production by CD4 T cells than the Breg counterparts from naïve mice (Fig. 3h). Treatment of tumor-bearing mice with FGK further enhanced the ability of these Bregs to suppress T cell activation, but to a significantly less magnitude in mice receiving GSK (Fig. 3h). These results indicate that PPARδ also plays a key role in tumor-induced development and/or maintenance of IL-10^+^ Bregs and their IL-10 secretion and immunosuppressive activity. Furthermore, engagement with agonistic anti-CD40 antibody may stimulate IL-10^+^ Bregs, leading to an increase in number and function in tumor-bearing mice by a mechanism largely dependent on PPARδ.

We also examined Bregs in the tumor and found that tumor-infiltrating Bregs, like lymph node Bregs, could not be clearly divided into CD24^hi^IgD^lo/−^CD38^lo^ and CD24^hi^IgD^lo/−^CD38^hi^ subsets (Supplementary Fig. S9a), but also expressed a higher level of PPARδ and produced more IL-10 (though not as high as splenic and blood Bregs; Fig. 2e–g and Supplementary Fig. S5b, c) than mature (CD24^lo^IgD^hi^) B cells (Supplementary Fig. S9b, c). Furthermore, GSK treatment resulted in a significant decrease in tumor-infiltrating Bregs in both control (Rat IgG-treated) and FGK-treated mice (Supplementary Fig. S9d). However, it should be noted that Bregs only accounted for a very small proportion of tumor-infiltrating lymphocytes in all groups of mice (Supplementary Fig. S9d).

### PPARδ inactivation in B cells impairs the development and function of IL-10^+^ Bregs

**Fig. 4:**
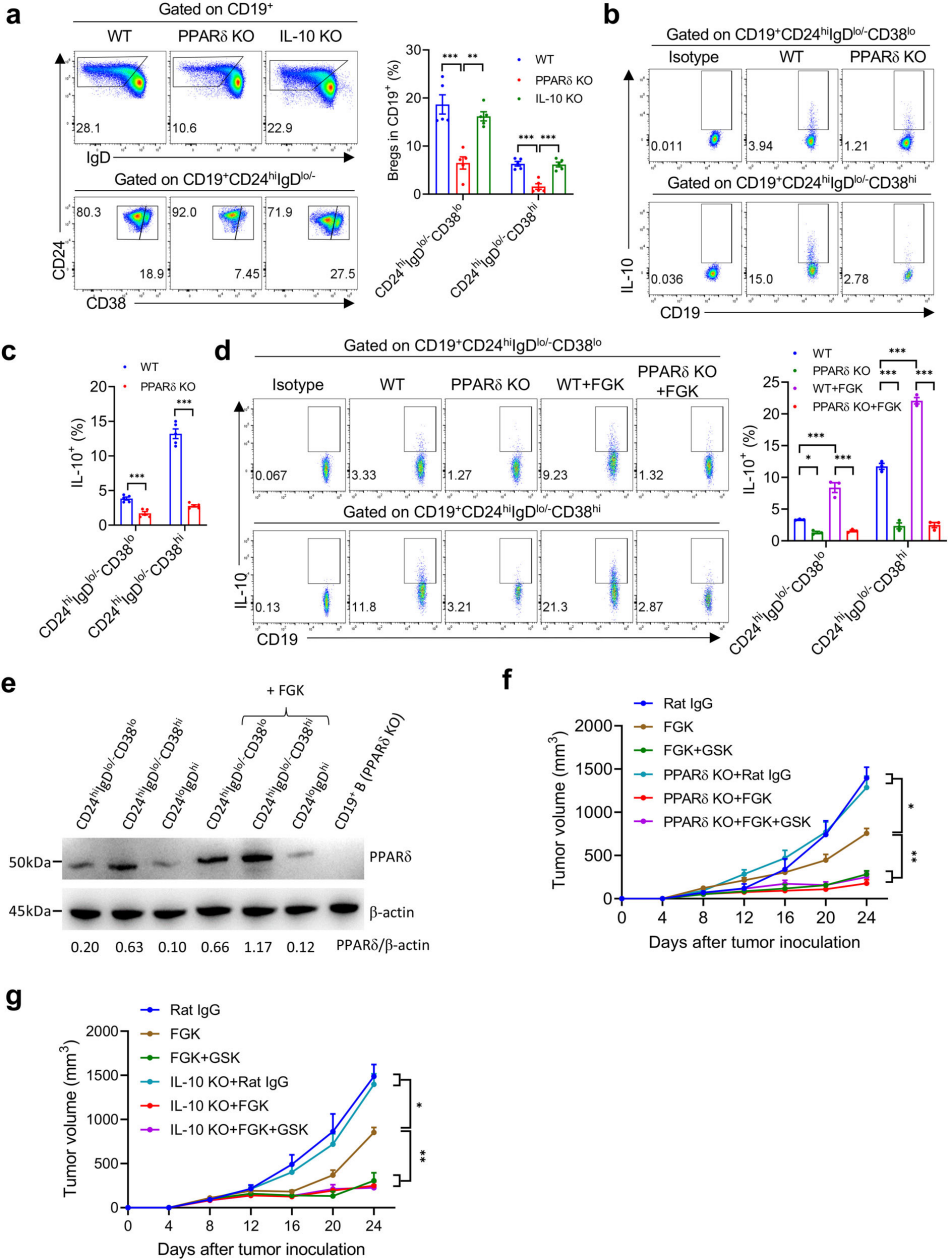
Impaired Breg development and function in *Cd19*^*Cre/Cre*^*Ppard*^*f/f*^ mice. **a** Representative profiles showing CD24, IgD and CD38 expression on gated CD19^+^ cells (left) and percentages of CD19^+^CD24^hi^IgD^lo/−^CD38^lo^ and CD19^+^CD24^hi^IgD^lo/−^CD38^hi^ Bregs in splenic B cells of naive, *Cd19*^*Cre/Cre*^*Ppard*^*f/f*^ (PPARδ KO) and *Cd19*^*Cre/Cre*^*IL-10*^*f/f*^ (IL-10 KO) mice (right; *n* = 5). **b**, **c** Representative profiles showing IL-10 staining (**b**) and percentages of IL-10^+^ cells (**c**) in the indicated splenic Breg subsets from naïve and *Cd19*^*Cre/Cre*^*Ppard*^*f/f*^ mice. **d**, **e** Splenic CD19^+^CD24^hi^IgD^lo/−^CD38^lo^ and CD19^+^CD24^hi^IgD^lo/−^CD38^hi^ Bregs were sorted from naïve WT and *Cd19*^*Cre/Cre*^*Ppard*^*f/f*^ mice, cultured without or with FGK (20 μg/mL) for 24 h, and examined for IL-10 secretion by flow cytometry (**d**) and PPARδ expression by western blot assay (**e**). **f**, **g** WT, *Cd19*^*Cre/Cre*^*Ppard*^*f/f*^ and *Cd19*^*cre/cre*^*IL-10*^*f/f*^ mice were inoculated s.c. with 7 × 10^5^ B16 cells and treated 8 days later with FGK or Rat IgG (peritumorally s.c.; 25 μg per day at day 8, 11 and 14), or FGK plus GSK (i.p, 300 nmol daily from day 8 to 16). **f** Tumor growth in WT vs. *Cd19*^*Cre/Cre*^*Ppard*^*f/f*^ mice (*n* = 6). **g** Tumor growth in WT vs. *Cd19*^*cre/cre*^*IL-10*^*f/f*^ mice (*n* = 6–7). Data are means ± SEM, or representative staining profiles or photographs. *P* values were determined by one-way ANOVA with Tukey’s multiple comparisons test (**a**, **d**), unpaired two-tailed Student’s *t* test (**c**), or two-way ANOVA with Tukey’s multiple comparisons test (**f**, **g**). Shown are data from a representative of two independent experiments (**a**–**g**).

Cd19^Cre/Cre^Ppard^f/f^ and Cd19^Cre/Cre^IL-10^f/f^ mice, with PPARδ and IL-10 deletion limited in CD19^+^ B cells, respectively, were used to further define the role of PPARδ in the development, maintenance and function of IL-10^+^ Bregs. Flow cytometric analysis revealed that Cd19^Cre/Cre^Ppard^f/f^ mice exhibited a significant reduction in CD24^hi^IgD^lo/−^CD38^lo^ and CD24^hi^IgD^lo/−^CD38^hi^ Bregs, in particular the latter population, compared to WT mice (Fig. 4a and Supplementary Fig. S10). Although frequencies of CD24^hi^IgD^lo/−^CD38^lo^ and CD24^hi^IgD^lo/−^CD38^hi^ B cells in Cd19^Cre/Cre^IL-10^f/f^ mice were comparable to WT mice (Fig. 4a and Supplementary Fig. S10), these B cells had no detectable inhibition on T cell activation (Supplementary Fig. S11). Furthermore, both Breg populations from Cd19^Cre/Cre^Ppard^f/f^ mice produced significantly less IL-10 than the counterparts of WT mice (Fig. 4b, c). Engagement with FGK resulted in a significant elevation in IL-10 secretion, which was associated with an increase in PPARδ expression, by WT CD24^hi^IgD^lo/−^CD38^lo^ and CD24^hi^IgD^lo/−^CD38^hi^ Bregs, while, in contrast, FGK failed to stimulate IL-10 secretion by Cd19^Cre/Cre^Ppard^f/f^ Bregs (Fig. 4d, e).

We then compared the antitumor effect of FGK, either alone or combined with GSK, in B16 tumor-bearing WT and Cd19^Cre/Cre^Ppard^f/f^ mice. Again, significantly improved antitumor responses were seen in WT mice treated with FGK plus GSK compared to those receiving FGK alone (Fig. 4f). However, FGK alone induced a significantly improved antitumor response in Cd19^Cre/Cre^Ppard^f/f^ mice (comparable to that in WT mice treated with FGK plus GSK), which was not further enhanced by GSK (Fig. 4f). Similar results were observed in Cd19^Cre/Cre^IL-10^f/f^ mice, in which FGK alone induced a strong antitumor response comparable to that in WT mice treated with FGK plus GSK, and no further enhancement was seen when treated with FGK plus GSK (Fig. 4g). Together, these results confirm that the effect of GSK treatment on antitumor immunity is mediated through targeting PPARδ^hi^ IL-10^+^ Bregs by inhibiting their PPARδ and hence IL-10 production.

### PPARδ inhibition blocks immunosuppressive function of tumor-induced IL-10^+^ Bregs in cancer patients

**Fig. 5:**
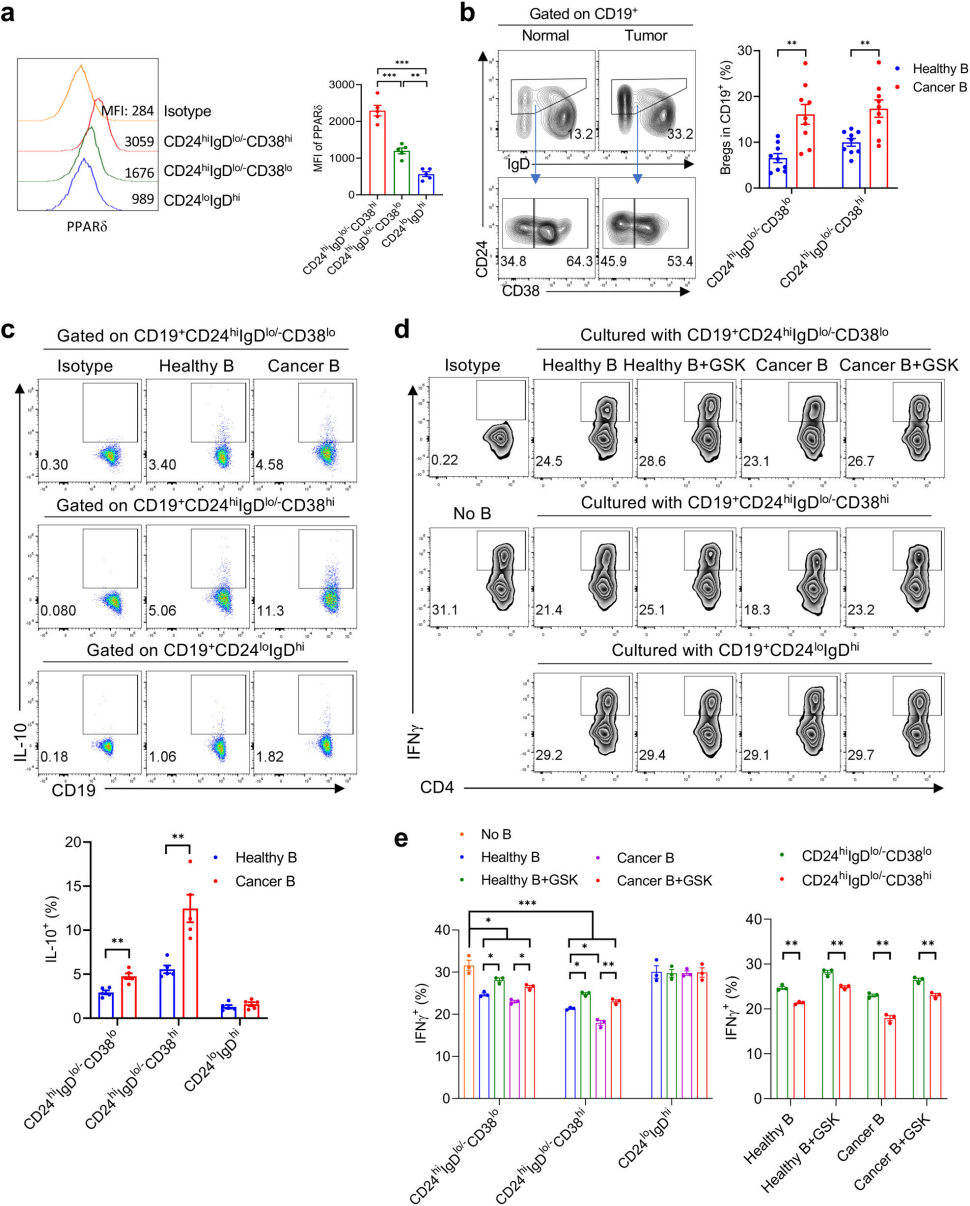
PPARδ inhibition blocks IL-10 secretion and immunosuppressive function of human IL-10^+^ Bregs. **a** Representative FACS profiles (left) and levels (MFI; right) of PPARδ expression in gated CD19^+^CD24^hi^IgD^lo/−^CD38^lo^, CD19^+^CD24^hi^IgD^lo/−^CD38^hi^ and CD19^+^CD24^lo^IgD^hi^ B cells from peripheral blood of healthy subjects (*n* = 5). **b** Representative profiles showing CD24, IgD and CD38 expression on gated CD19^+^ B cells (left) and percentages of CD19^+^CD24^hi^IgD^lo/−^CD38^lo^ and CD19^+^CD24^hi^IgD^lo/−^CD38^hi^ Bregs (right) in peripheral blood of healthy subjects and NSCLC patients (*n* = 9). **c** Representative profiles showing IL-10 staining (top panel) and percentages of IL-10^+^ cells (bottom) in the indicated B cell populations. **d**, **e** CD19^+^CD24^hi^IgD^lo/−^CD38^lo^, CD19^+^CD24^hi^IgD^lo/−^CD38^hi^ and CD19^+^CD24^lo^IgD^hi^ B cells were sorted from PBMCs of healthy subjects (*n* = 4) or NSCLC patients (*n* = 5) and pooled, respectively. The pooled cells were then cultured with or without GSK (200 nM) for 24 h, and examined for the ability to inhibit human CD4^+^CD25^−^ T cell activation in response to CD3/CD28 stimulation. Shown are representative FACS profiles (**d**) and percentages (**e**; *n* = 3; the left and right panels were analyzed with the same data) of IFNγ^+^ cells in CD4^+^ T cells. All data are means ± SEM, and *P* values were determined by one-way ANOVA with Tukey’s multiple comparisons test (**a**, **e** left) or unpaired two-tailed Student’s *t* test (**b**, **c**, **e** right). Data from a representative of two (**a**) or three (**c**–**e**) independent experiments are shown.

We further validated the clinical relevance of our findings in non-small-cell lung cancer (NSCLC) patients with adenocarcinoma or squamous cell carcinoma (Supplementary Table S1). Similar to the observations in mice, human CD19^+^CD24^hi^IgD^lo/−^CD38^lo^ and CD19^+^CD24^hi^IgD^lo/−^CD38^hi^ Bregs were also found to express a higher level of PPARδ than human CD19^+^CD24^lo^IgD^hi^ mature B cells (Fig. 5a), and frequencies of both Breg subsets were increased in cancer patients compared to healthy subjects (Fig. 5b). Furthermore, both Breg subsets, in particular the CD24^hi^IgD^lo/−^CD38^hi^ Breg subset, of the cancer patients consisted of significantly more IL-10^+^ cells than the counterparts of healthy subjects (Fig. 5c). However, the level of IL-10 expression in CD19^+^CD24^lo^IgD^hi^ mature B cells was comparably low in normal subjects and patients (Fig. 5c). Importantly, CD19^+^CD24^hi^IgD^lo/−^CD38^lo^ and CD19^+^CD24^hi^IgD^lo/−^CD38^hi^ Bregs from cancer patients showed a significantly stronger ability to inhibit T cell activation (as measured by IFNγ production) than those from healthy subjects (Fig. 5d, e, left). Furthermore, the inhibitory activity of human CD19^+^CD24^hi^IgD^lo/−^CD38^lo^ and CD19^+^CD24^hi^IgD^lo/−^CD38^hi^ Bregs from both healthy subjects and cancer patients was significantly reduced by treatment with GSK (Fig. 5d, e, left). Of note, CD19^+^CD24^hi^IgD^lo/−^CD38^hi^ Bregs from peripheral blood of both healthy subjects and cancer patients showed stronger suppression than CD19^+^CD24^hi^IgD^lo/−^CD38^lo^ Bregs (Fig. 5e, right).

We also examined peripheral blood samples of patients with ovarian cancer or urothelial cancer (Supplementary Tables S2 and S3). The proportion of CD19^+^CD24^hi^IgD^lo/−^CD38^lo^ Bregs in ovarian cancer patients was comparable to that in healthy subjects, but the proportion of CD19^+^CD24^hi^IgD^lo/−^CD38^hi^ Bregs in ovarian cancer patients was increased (Supplementary Fig. S12a). A significant increase in both CD19^+^CD24^hi^IgD^lo/−^CD38^lo^ and CD19^+^CD24^hi^IgD^lo/−^CD38^hi^ Bregs was found in urothelium cancer patients compared to healthy subjects (Supplementary Fig. S12a). Furthermore, Bregs in both groups of patients exhibited a significantly increased PPARδ expression (Supplementary Fig. S12b, c) and IL-10 production (Supplementary Fig. S12d) compared to the counterpart Bregs of healthy subjects. Together, these results indicate that, consistent with the findings in mice, human IL-10^+^ Bregs also highly express PPARδ, and their expansion and function are largely PPARδ dependent.

## Discussion

In this study, we report that PPARδ is highly expressed in IL-10^+^ Bregs with a predominant surface phenotype of CD19^+^CD24^hi^IgD^lo/−^CD38^lo^ or CD19^+^CD24^hi^IgD^lo/−^CD38^hi^, which present in naïve mice and can be induced by tumor formation and agonistic anti-CD40 mAb. Using mice with PPARδ inactivation in B cells, we confirmed that PPARδ signaling is essential for the development and immunosuppressive activity of IL-10^+^ Bregs. PPARδ inactivation not only significantly reduced IL-10^+^ Bregs in naïve mice, but also prevented IL-10^+^ Breg induction by tumor and anti-CD40 engagement. Furthermore, treatment of tumor-bearing mice with PPARδ inhibitor could efficiently diminish the number and function of IL-10^+^ Bregs, and act synergistically with anti-CD40 or anti-PD1 antibody to improve antitumor immune responses. These findings provide direct evidence supporting the potential of PPARδ as a target for selectively inhibiting tumor-induced Bregs to aid cancer immunotherapy.

Increasing evidence demonstrates that Bregs are powerful in suppressing antitumor responses, and considered an important factor limiting the efficacy of cancer immunotherapies^5–7^. However, it remains challenging to develop practical strategies to specifically eliminate Bregs due to the lack of a druggable marker or target that can identify Bregs and distinguish them from non-Breg B cell populations^12^. Because of the highly functional heterogeneity of B cells, and some are capable of eliciting antitumor immunity^8–12^, pan-B cell depletion may result in unwanted outcomes. Previous studies have shown that B cell depletion could be effective^5,6^, ineffective^13,14^, or even detrimental^15^ regarding to the effect on antitumor responses. Our results showed that PPARδ inhibitor is efficient in inhibiting tumor- and CD40 agonist-induced IL-10^+^ Bregs, and preventing transition of CD19^+^CD24^hi^IgD^lo/−^CD38^lo^ Bregs to CD19^+^CD24^hi^IgD^lo/−^CD38^hi^ Bregs with increased expression of PPARδ and IL-10, leading to markedly improved antitumor responses. Although PPARδ is expressed by multiple types of cells, including macrophages^18,19^ and T cells^33^, the expression levels were considerably lower than IL-10^+^ Bregs (Supplementary Fig. S13). Previous studies using mice with genetic PPARδ deletion revealed that PPARδ deficiency impairs M2 differentiation and function^18,19^, as well as thymocyte development and differentiation^33^. However, alterations in PPARδ expression or function were not detectable in macrophages or T cells in mice treated with GSK at doses that could effectively inhibit IL-10^+^ Bregs (Supplementary Figs. S3c and S14). These findings suggest that the level of PPARδ expression is a crucial factor determining the sensitivity of cells to PPARδ inhibitor.

Although treatment with GSK was found to inhibit Bregs, it did not significantly inhibit tumor growth when used alone, indicating that GSK may not significantly improve the intratumoral immune response or destruction of tumor cells. In agreement with this, we found that Bregs only accounted for a very small proportion (∼0.2%) of tumor-infiltrating immune cells, suggesting that tumor is not the main site for Bregs to inhibit antitumor immune responses. LNs are the main site of T cell activation by antigens, and a good antitumor response requires a complete LN structure^34^. Furthermore, the proportion of Bregs to T cells in LNs is much higher than in tumor. Thus, we propose that dLN is an important site where Bregs inhibit the induction of antitumor immune responses, which may explain why GSK treatment could act synergistically with anti-CD40 antibodies or immune checkpoint blockades to improve antitumor effects, but failed to do so when used alone.

Although the role of B cells in human cancer immunity and immunotherapy remains poorly understood, the presence of IL-10^+^ Bregs in human cancers was reported previously, and their presence was found, for some cancer types, to be an indicator of poor prognosis^12,32^. Here, we found that patients with NSCLC, ovarian or urothelial cancer also had a significant increase in IL-10^+^ Bregs than healthy subjects. Importantly, these Bregs also expressed significantly higher levels of PPARδ than mature B cells, and that the level of PPARδ expression positively correlated with their potential to produce IL-10. Furthermore, the ability of these Bregs to produce IL-10 and to suppress T cell activation was significantly suppressed by treatment with PPARδ inhibitor. Thus, PPARδ offers a potentially druggable target for inhibiting human IL-10^+^ Bregs.

## Materials and methods

### Animals

C57BL/6N mice of 6–8 weeks of age were purchased from Charles River (Beijing, China). C57BL/6J *Cd19*^*Cre*^, C57BL/6J *Ppard*^*flox*^, C57BL/6J *IL-10*^*flox*^ mice were purchased from Shanghai Model Organisms, and *Cd19*^*Cre/Cre*^*Ppard*^*f/f*^ and *Cd19*^*Cre/Cre*^*IL-10*^*f/f*^ mice were generated by breeding C57BL/6J *Cd19*^*Cre*^ mice with C57BL/6J *Ppard*^*flox*^ mice or with C57BL/6J *IL-10*^*flox*^ mice, respectively. Protocols involving the use of animals were reviewed and approved by the Institutional Animal Care and Use Committee of the First Hospital of Jilin University and all experiments were performed in accordance with the protocols.

### Production of PPARγ-GFP reporter mice

PPARγ-GFP reporter mice were established using CRISPR/Cas9 technology. Briefly, a vector containing Cas9 and sgRNA sequences (5’-tagaaggaacacgttgtcagcgg-3’) recognizing the 3’ end of exon 7 (gene id:19016), and a targeting vector containing IRES-GFP (Addgene) flanked by 1.7 kb 5’ and 1.1 kb 3’ homologous arms were constructed and microinjected into fertilized eggs of C57BL/6 mice. IRES-GFP was inserted between the 3’ end of exon 7 and 3’-UTR of *pparg* gene. The genotypes of F0 mice were screened by PCR and sequencing. The F1 generation of the PPARγ-GFP reporter mice with stable genotype were established by mating F0 mice with wild type C57BL/6 mice, and identified by PCR and Southern blot analysis with 5’ probe and GFP probe. The sequences of PCR primes are 5’-taaacggccacaagttcagc-3’ and 5’-aagataaccttggccaggcagt-3’.

### Abs and reagents

The following anti-mouse monoclonal antibodies (mAbs) used for cell surface or intracellular staining were obtained from Biolegend (San Diego, CA): FITC-anti-CD45 (clone 30-F11), PB-anti-CD45 (clone 30-F11), PE/Dazzle 594-anti-CD3 (clone 17A2), PE/Cy7-anti-CD3 (clone 17A2), PE-Cy5-anti-CD8a (53-6.7), BV605-anti-CD8a (clone 53-6.7), Percp/Cy5.5-anti-CD4 (clone GK1.5), AF647-anti-F4/80 (clone BM8), APC/Cy7-anti-CD11b (clone M1/70), PE-anti-CD11c (clone N418), APC-anti-CD19 (clone 6D5), APC/Cy7-anti-CD19 (clone 6D5), BV650-anti-CD19 (clone 6D5), APC/Cy7-anti-PD1 (clone 29F.1A12), APC-anti-Granzyme B (clone QA16A02), PE-anti-perforin (clone S16009A), BV711-anti-CD107a (clone 1D4B), BV421-anti-CD24 (clone M1/69), FITC-anti-CD24 (clone M1/69), APC-anti-IgD (clone 11-26c.2a), BV510-anti-IgD (clone 11-26c.2a), PE/Cy7-anti-CD38 (clone 90), PE-anti-CD38 (clone 90), PE/Cy5-anti-IgM (clone RMM-1), BV711-anti-CD5 (clone 53-7.3), BV650-anti-CD138 (clone 281-2), PB-anti-CD1d (clone 1B1), BV570-anti-CD45R (clone RA3-6B2), BV605-anti-CD23 (clone B3B4), AF700-anti-CD21/35 (clone 7E9), APC/Cy7-anti-CD27 (clone LG.3A10), PE-anti-CD27 (clone LG.3A10), BV785-anti-CD274 (clone 10F.9G2), Percp/Cy5.5-anti-CD93 (clone AA4.1), PE-anti-CD365 (clone RMT1-4), PE-anti-CD20 (clone SA271G2), APC-anti-IFNγ (clone XMG1.2), Percp/Cy5.5-anti-IFNγ (clone XMG1.2), PE/Dazzle 594-anti-IL-10 (clone JES5-16E3), AF700-anti-Ki67 (clone 16A8), APC-anti-Rat IgG1, κ Isotype (clone RTK2071), Percp/Cy5.5-anti-Rat IgG1, κ Isotype (clone RTK2071), PE/Dazzle 594-anti-Rat IgG2b, κ Isotype (clone RTK4530), PE-anti-Rat IgG2a, κ Isotype (clone RTK2758) and AF700-anti-Rat IgG2a, κ Isotype (clone RTK2758). BV480-anti-CD45 (clone 30-F11), PE-anti-CD4 (clone H129.19) and FITC-anti-Mouse IgG1, κ Isotype (clone MOPC-21) were purchased from BD Biosciences (Mountain View, CA). PE-anti-CD21/35 (clone 8D9) and APC-anti-IgA (clone mA-6E1) were purchased from Invitrogen (Carlsbad, CA). FITC-anti-mouse/human PPARβ/δ (clone F-10) was purchased from Santa Cruz (Santa Cruz, CA).

The following anti-human mAbs used for cell surface or intracellular staining were obtained from Biolegend (San Diego, CA): APC/Cy7-anti-CD19 (clone HIB19), APC-anti-CD19 (clone HIB19), FITC-anti-CD24 (clone ML5), BV785-anti-CD24 (clone ML5), PE/Cy7-anti-CD38 (HB-7), AF647-anti-IgD (clone IA6-2), Percp/Cy5.5-anti-IgD (clone IA6-2), AF700-anti-IgM (MHM-88), APC/Cy7-anti-CD5 (clone UCHT2), BV570-anti-CD45R (clone RA3-6B2), PE-anti-CD27 (LG.3A10), APC/Cy7-anti-CD27 (LG.3A10), APC-anti-CD21 (clone Bu32), PB-anti-CD138 (clone MI15), APC/Cy7-anti-CD4 (clone OKT4), PE/Cy5-anti-CD4 (clone OKT4), AF647-anti-CD25 (clone BC96), Percp/Cy5.5-anti-IFNγ (clone 4S.B3), BV421-anti-IL-10 (clone JES3-9D7), Percp/Cy5.5-anti-mouse IgG1, κ Isotype (clone MOPC-21), BV421-anti-Rat IgG1, κ Isotype (clone RTK2071) and AF647-anti-mouse IgG1, κ Isotype (clone MOPC-21). BV480-anti-CD45 (clone HI30), BV711-anti-CD3 (clone UCHT1), BV605-anti-CD8 (clone SK1), BV711-anti-CD19 (clone SJ25C1), Percp/Cy5.5-anti-CD138 (clone MI15) and AF647-anti-IFNγ (clone 4S.B3) were purchased from BD Biosciences (Mountain View, CA). Percp-eFlour 710-anti-CD1d (clone 51.1) and PE-eFluor610-anti-CD23 (clone EBVCS2) were purchased from Invitrogen (Carlsbad, CA).

Antibodies for in vivo treatments include agonist anti-mouse CD40 (clone FGK45.5 or FGK) mAb, Rat IgG2a (clone 2A3) and anti-CD19 mAb (clone 1d3), which were purchased from BioXcell (West Lebanon, NH). LIVE/DEAD™ Fixable Aqua Dead Cell Stain Kit (catalog number: L34957) and CellTrace CFSE Cell Proliferation Kit (catalog number: C34574) were purchased from Invitrogen (Carlsbad, CA). PPARγ inhibitor T0070907 (T007; catalog number: S2871) and PPARδ inhibitor GSK3787 (GSK; catalog number: S8025) were purchased from Selleck. Dynabeads-coated with anti-human or mouse mAb to CD3 plus mAb to CD28 (catalog number: 11131D or 11456D) were purchased from GIBCO BRL (Grand Island, NY).

### Cell staining and flow cytometry

For cell surface staining, single-cell suspensions were suspended in FACS buffer (PBS containing 0.5% BSA) and stained with fluorochrome-conjugated Abs on ice for 30 min. The cells were washed twice with FACS buffer and fixed in 1% paraformaldehyde for FACS analysis. For mouse IL-10 staining, mouse B cells were stimulated for 5 h with phorbol 12-myristate 13-acetate (50 ng/mL), ionomycin (550 ng/mL; Sigma-Aldrich) and LPS (1 μg/mL) in the presence of GolgiPlug (BD Biosciences). For human IL-10 staining, human B cells were stimulated for 48 h with 2.5 mg/mL CpG-B ODN 2006 from Invivogen and 0.5 mg/mL recombinant human CD40L from Biolegend. Cells were further stimulated for 5 h with PMA (50 ng/mL) and ionomycin (550 ng/mL) in the presence of GolgiPlug before staining. For mouse/human IFNγ and mouse granzyme B, perforin staining, T cells were stimulated for 5 h with PMA (50 ng/mL) and ionomycin (550 ng/mL) in the presence of GolgiPlug. Then cells were fixed and made permeable with Cytofix/Cytoperm solution (BD Biosciences) and were stained with anti-mouse IL-10-PE/Dazzle 594 (clone JES5-16E3; Biolegend), anti-human IL-10-BV421 (clone JES3-9D7; Biolegend), anti-mouse IFNγ-APC (clone XMG1.2; Biolegend) or anti-human IFNγ-AF647 (clone 4S.B3; BD Biosciences), anti-mouse granzyme B-APC(clone QA16A02; Biolegend) or anti-mouse perforin-PE(clone S16009A; Biolegend) according to the manufacturers’ instructions. For PPARδ intracellular staining, mouse B cells, mouse macrophages or human B cells were stained firstly for surface antigens and washed twice with FACS buffer, then fixed and permeabilized with foxp3/transcription factor staining buffer set (eBioscience), followed by staining with anti-mouse/human PPARβ/δ-FITC mAb (clone F-10; Santa Cruz) according to the manufacturers’ instructions. For Ki67 intracellular staining, mouse B cells were stained firstly for surface antigens and washed twice with FACS buffer, then fixed and permeabilized with foxp3/transcription factor staining buffer set, followed by staining with anti-Ki67-Alexa Fluor 700 (clone 16A8; Biolegend). All samples were acquired using BD LSR Fortessa (BD Biosciences, Mountain View, CA) or Cytek Aurora (Cytek Biosciences, Fremont, CA) and data analysis was performed using FlowJo software (Tree Star).

### t-SNE analysis

Spleen cells from WT, *Cd19*^*Cre/Cre*^*Ppard*^*f/f*^ (PPARδ KO) and *Cd19*^*Cre/Cre*^*IL-10*^*f/f*^ (IL-10 KO) mice were stained with multiple markers including CD45, CD19, B220, CD1d, IgM, CD5, CD23, IgD, CD21/35, CD27, CD138, CD38, CD24, IL-10 and PPARδ, and analyzed by Cytek Aurora. To generate t-SNE maps, flow cytometry data were first gated for live CD45^+^CD19^+^ B cells, and then randomly down sampled to appropriate events for each sample (200,000/sample for Fig. 2; 20,000/sample for Supplementary Fig. S10) and the resulting data concatenated into a single dataset. t-SNE was used to reduce dimensionality of the dataset with the following settings: Auto(opt-SNE), Iterations: 1000, KNN algorithm: Exact (vantage point tree), gradient algorithm: Barnes-Hut. Then the automated clustering analysis was done by the FlowSOM algorithm for clustering of the data into subpopulations. The resulting subpopulations were further analyzed for specific marker expression and frequency. All data were analyzed using FlowJo version 10.

### Western blot analysis

In total, 2 × 10^6^ cells were pelleted and collected in 1.5 mL EP tubes and lysed on ice for 15 min with 100 μL RIPA lysis buffer (Invitrogen, P0013E). The samples were then centrifuged at 12,000 rpm for 10 min at 4 °C, and the supernatant was transferred to a new EP tube. After the concentration was detected by Pierce™ BCA Protein Assay Kit (Thermo Fisher Scientific, Cat# 23227), the samples were denatured by boiling at 100 °C for 10 min.

Fifty μg of total protein from each sample was loaded for sodium dodecyl sulfate-polyacrylamide gel electrophoresis analysis. Protein samples were loaded onto 4%–12% precast gels (Beyotime, P0056A) for electrophoresis, and then the gels were blotted to nitrocellulose membranes. Nitrocellulose membranes were blocked with 5% skim milk in Tris-buffered saline with Tween-20 (TBST, 10 mM Tris pH 8.0, 150 mM NaCl and 0.1% Tween 20) for 1 h at room temperature. Membranes were then incubated overnight with the primary anti-PPARδ antibody (1:1000 dilution, Abcam, ab137724) or anti-β-actin antibody (1:1000 dilution, Cell Signaling Technology, 3700S) in 5% BSA-TBST buffer. Membranes were washed and incubated with anti-rabbit/mouse IgG, HRP-linked Antibody (1:5000 dilution; Cell Signaling Technology, 7074/7076) for 1 h at room temperature. After washing, the membrane was visualized with High-sig ECL Western Blotting Substrate (Tanon, 180-5001), and the bands were quantified with ImageJ software.

### Tumor cell lines and culture

The murine cell lines of B16 melanoma and MB49 bladder cancer were obtained from the American Type Culture Collection. The above cells were maintained in DMEM (Carlsbad, CA, USA) supplemented with 10% FBS (Waltham, MA, USA), 100 U/mL penicillin and 100 U/mL streptomycin. All cells were cultured in a humidified incubator at 37 °C, with 5% CO_2_.

### Tumor models and treatments

B16 or MB49 tumor cells were washed and resuspended in PBS and s.c. injected (7 × 10^5^ cells/mouse in 100 mL PBS) into the flank of mice which had previously shaved. The mice were monitored every day and tumors were measured with a caliper every 4 days. Tumor volumes were measured by length (*a*) and width (*b*) and calculated as tumor volume = *ab*^2^/2. When tumor reached about 4–5 mm in diameter (i.e., between 6 and 8 days after B16 or MB49 cells injection), the animals were randomly divided into groups and treated with peritumoral injection (s.c.) of FGK or isotype-matched antibody (Rat IgG2a; clone 2A3), and/or intraperitoneal injection of T007 or GSK at the indicated dosage and schedule. Unless otherwise indicated, both T007 (a selective PPARγ inhibitor with IC_50_ of 1 nM) and GSK (a selective and irreversible PPARδ antagonist with IC_50_ of 6.6 nM) were given at ~45 times of their IC_50_ (i.e., 45 nM and 300 nM, respectively). In the experiments assessing antitumor immune memory, mice surviving first tumor challenge were rechallenged by B16 melanoma cells using the same number of cells as the first time via tail vein injection. For in vivo depletion of CD19^+^ B cells, mice were injected with 250 μg anti-CD19 antibody (clone 1d3; intraperitoneal, i.p.) at days 6, 7 and 15 after inoculation of tumor cells. Efficient depletion was confirmed by measuring CD20^+^ cells in blood and tissues. The mice were euthanized at the indicated timepoints or when tumor volume reached 1500 mm^3^.

### Serum ALT analysis

Serum samples were taken from mice as the indicated time points, and measured for ALT levels using an alanine aminotransferase Assay Kit (Nanjing Jiancheng Bioengineering Institute, C009-2) according to the manufacturers’ instructions.

### Tissue histopathology and IHC analysis

Liver and tumor were prepared from mice at the indicated time points after treatment, fixed with 4% paraformaldehyde and embedded in paraffin, and sectioned for H&E (2.5 μm) and IHC (4 μm) staining. For IHC staining, tissue sections were stained with monoclonal rabbit anti-mouse CD8a antibody (Abcam, ab209775), and the immunoreactivity was detected with UltraSensitiveTM Streptavidin-Peroxidase Kit (Mai Xin, KIT-9706) according to the manufacturers’ protocol. Images were captured using an IX2-SL microscope and processed using CellSens Dimension software.

### Tissue harvest and dissociation

For cell isolation from tumor tissues, tumors were collected in ice-cold PBS and diced into small pieces (around 1 mm^3^) and incubated with digestive solution (0.1% (w/v) type IV collagenase) at 37 °C for 30 min in a water bath shaker. Spleens and inguinal lymph nodes were harvested from normal or tumor-bearing (the tumor dLNs) mice, crushed, and single-cell suspensions were obtained by filtering through a 70 μm cell strainer (Millipore).

### In vitro mouse B cell suppression assay

Naïve mouse CD4^+^CD25^−^ or CD8^+^ T cells were sorted from spleens of 6–8-week-old C57BL/6 mice using BD Influx TM (BD Biosciences). CD19^+^ cells from dLNs, CD19^+^CD24^hi^IgD^lo/−^CD38^lo^, CD19^+^CD24^hi^IgD^lo/−^CD38^hi^ and CD19^+^CD24^lo^IgD^hi^ B cells from spleens of naïve or tumor-bearing mice were sorted. Purified B cells were cocultured with T cells from naïve mice (at 1:1 ratio) in the presence of anti-mouse CD3/CD28 dynabeads in 96-well plates with U-shaped bottom for 3 days. GolgiPlug (BD Biosciences) was added for the last 5 h along with PMA (50 ng/mL) and ionomycin (550 ng/mL). T cell function was analyzed by measuring IFNγ secretion using flow cytometry.

### Mouse T cells proliferation assay

Purified CD4 or CD8 T cells were labeled with 2.5 μM CFSE ex vivo. The stained cells were resuspended in complete RPMI 1640 medium to 1 × 10^6^/ml and cultured with or without 300 nm GSK3787 in the presence of anti-mouse CD3/CD28 dynabeads in 96-well plates with U-shaped bottom for 72 h. T cell proliferation was analyzed by measuring CFSE dilution using flow cytometry. Cell proliferation rate of CFSE-labeled T cells was calculated using the following formula: (1 − MFI value of CFSE of T cells in experimental group/MFI value of CFSE of T cells in control group) × 100%.

### Human samples

Blood samples were obtained from cancer patients with adenocarcinoma, squamous cell carcinoma, ovarian cancer or urothelial cancer (diagnosed by imaging and histopathology; Supplementary Tables S1–S3) and healthy individuals (aged 45–60 years), and PBMCs were isolated by Ficoll-Paque Plus (Amersham Biosciences, Uppsala, Sweden) gradient centrifugation. All participants were given written informed consent and all procedures in this study were approved (reference number: 2021-028) by the ethics committee of the First Hospital of Jilin University.

### In vitro human B cell suppression assay

B cells in peripheral blood of multiple healthy subjects or patients were isolated and pooled. CD19^+^CD24^hi^IgD^lo/−^CD38^lo^, CD19^+^CD24^hi^IgD^lo/−^CD38^hi^ and CD19^+^CD24^lo^IgD^hi^ B cells were sorted from the pooled B cells using BD Influx TM and cultured in complete RPMI 1640 medium with or without GSK for 24 h. Then, these B cells were washed twice, and cocultured with CD4^+^CD25^−^ T cells from healthy subjects (at 1:1 ratio) in the presence of anti-human CD3/CD28 dynabeads in 96-well plates with U-shaped bottom for 48 h. T cell function was analyzed by measuring IFNγ using flow cytometry.

### Statistical analysis

Statistical analyses were performed on GraphPad Prism 7 and data are presented as means ± SEM. *P* values were calculated using unpaired two-tailed Student’s *t* test and one-way analysis of variance (ANOVA) with Tukey’s multiple comparisons test. The statistical evaluation of mouse survival was performed using the log-rank test. Tumor growth curve was analyzed using two-way ANOVA with Tukey’s multiple comparisons test. *P* value of < 0.05 is considered significant.

## Supplementary information

Supplementary Information

## Data Availability

All data supporting the findings of this study are available within the main text and Supplementary Materials.

## References

[1] Ribas A, Wolchok JD (2018). Cancer immunotherapy using checkpoint blockade. Science.

[2] Yost KE (2019). Clonal replacement of tumor-specific T cells following PD-1 blockade. Nat. Med..

[3] Vonderheide RH (2020). CD40 agonist antibodies in cancer immunotherapy. Annu. Rev. Med..

[4] Teng MW, Ngiow SF, Ribas A, Smyth MJ (2015). Classifying cancers based on T-cell infiltration and PD-L1. Cancer Res..

[5] Shalapour S (2015). Immunosuppressive plasma cells impede T-cell-dependent immunogenic chemotherapy. Nature.

[6] Somasundaram R (2017). Tumor-associated B-cells induce tumor heterogeneity and therapy resistance. Nat. Commun..

[7] Glass MC (2022). Human IL-10-producing B cells have diverse states that are induced from multiple B cell subsets. Cell Rep..

[8] Lu Y (2020). Complement signals determine opposite effects of B cells in chemotherapy-induced immunity. Cell.

[9] Petitprez F (2020). B cells are associated with survival and immunotherapy response in sarcoma. Nature.

[10] Helmink BA (2020). B cells and tertiary lymphoid structures promote immunotherapy response. Nature.

[11] Cabrita R (2020). Tertiary lymphoid structures improve immunotherapy and survival in melanoma. Nature.

[12] Laumont CM, Banville AC, Gilardi M, Hollern DP, Nelson BH (2022). Tumour-infiltrating B cells: immunological mechanisms, clinical impact and therapeutic opportunities. Nat. Rev. Cancer.

[13] Aklilu M (2004). Depletion of normal B cells with rituximab as an adjunct to IL-2 therapy for renal cell carcinoma and melanoma. Ann. Oncol..

[14] Damsky W (2019). B cell depletion or absence does not impede anti-tumor activity of PD-1 inhibitors. J. Immunother. Cancer.

[15] Bodogai M (2013). Anti-CD20 antibody promotes cancer escape via enrichment of tumor-evoked regulatory B cells expressing low levels of CD20 and CD137L. Cancer Res..

[16] Mohib K, Cherukuri A, Rothstein DM (2018). Regulatory B cells and transplantation: almost prime time?. Curr. Opin. Organ Transpl..

[17] Odegaard JI (2007). Macrophage-specific PPARgamma controls alternative activation and improves insulin resistance. Nature.

[18] Kang K (2008). Adipocyte-derived Th2 cytokines and myeloid PPARdelta regulate macrophage polarization and insulin sensitivity. Cell Metab..

[19] Odegaard JI (2008). Alternative M2 activation of Kupffer cells by PPARdelta ameliorates obesity-induced insulin resistance. Cell Metab..

[20] Daniel B (2018). The nuclear receptor PPARgamma controls progressive macrophage polarization as a ligand-insensitive epigenomic ratchet of transcriptional memory. Immunity.

[21] Saha D, Martuza RL, Rabkin SD (2017). Macrophage polarization contributes to glioblastoma eradication by combination immunovirotherapy and immune checkpoint blockade. Cancer Cell.

[22] DeNardo DG, Ruffell B (2019). Macrophages as regulators of tumour immunity and immunotherapy. Nat. Rev. Immunol..

[23] Fransen MF, Sluijter M, Morreau H, Arens R, Melief CJ (2011). Local activation of CD8 T cells and systemic tumor eradication without toxicity via slow release and local delivery of agonistic CD40 antibody. Clin. Cancer Res..

[24] Zhang J, Li Y, Yang S, Zhang L, Wang W (2019). Anti-CD40 mAb enhanced efficacy of anti-PD1 against osteosarcoma. J. Bone Oncol..

[25] Ngiow SF (2016). Agonistic CD40 mAb-driven IL12 reverses resistance to anti-PD1 in a T-cell-rich tumor. Cancer Res..

[26] Knorr DA, Dahan R, Ravetch JV (2018). Toxicity of an Fc-engineered anti-CD40 antibody is abrogated by intratumoral injection and results in durable antitumor immunity. Proc. Natl. Acad. Sci. USA.

[27] Liu C (2020). Neuropilin-1 is a T cell memory checkpoint limiting long-term antitumor immunity. Nat. Immunol..

[28] Reading JL (2018). The function and dysfunction of memory CD8(+) T cells in tumor immunity. Immunol. Rev..

[29] Mauri C, Bosma A (2012). Immune regulatory function of B cells. Annu. Rev. Immunol..

[30] Matsumoto M (2014). Interleukin-10-producing plasmablasts exert regulatory function in autoimmune inflammation. Immunity.

[31] Michaud D, Steward CR, Mirlekar B, Pylayeva-Gupta Y (2021). Regulatory B cells in cancer. Immunol. Rev..

[32] Sharonov GV, Serebrovskaya EO, Yuzhakova DV, Britanova OV, Chudakov DM (2020). B cells, plasma cells and antibody repertoires in the tumour microenvironment. Nat. Rev. Immunol..

[33] Zhao FL (2018). Peroxisome proliferator-activated receptor-δ supports the metabolic requirements of cell growth in TCRβ-selected thymocytes and peripheral CD4(+) T cells. J. Immunol..

[34] Rahim MK (2023). Dynamic CD8(+) T cell responses to cancer immunotherapy in human regional lymph nodes are disrupted in metastatic lymph nodes. Cell.

