# Supplementary material for: PPARδ inhibition blocks the induction and function of tumor-induced IL-10+ regulatory B cells and enhances cancer immunotherapy
Source: Cell Discov. 2023 Jun 8;9:54. doi: 10.1038/s41421-023-00568-6 (PMC10250529; doi:10.1038/s41421-023-00568-6)
Supplement: Supplementary file 1 — Supplementary Information [file 41421_2023_568_MOESM1_ESM.pdf]

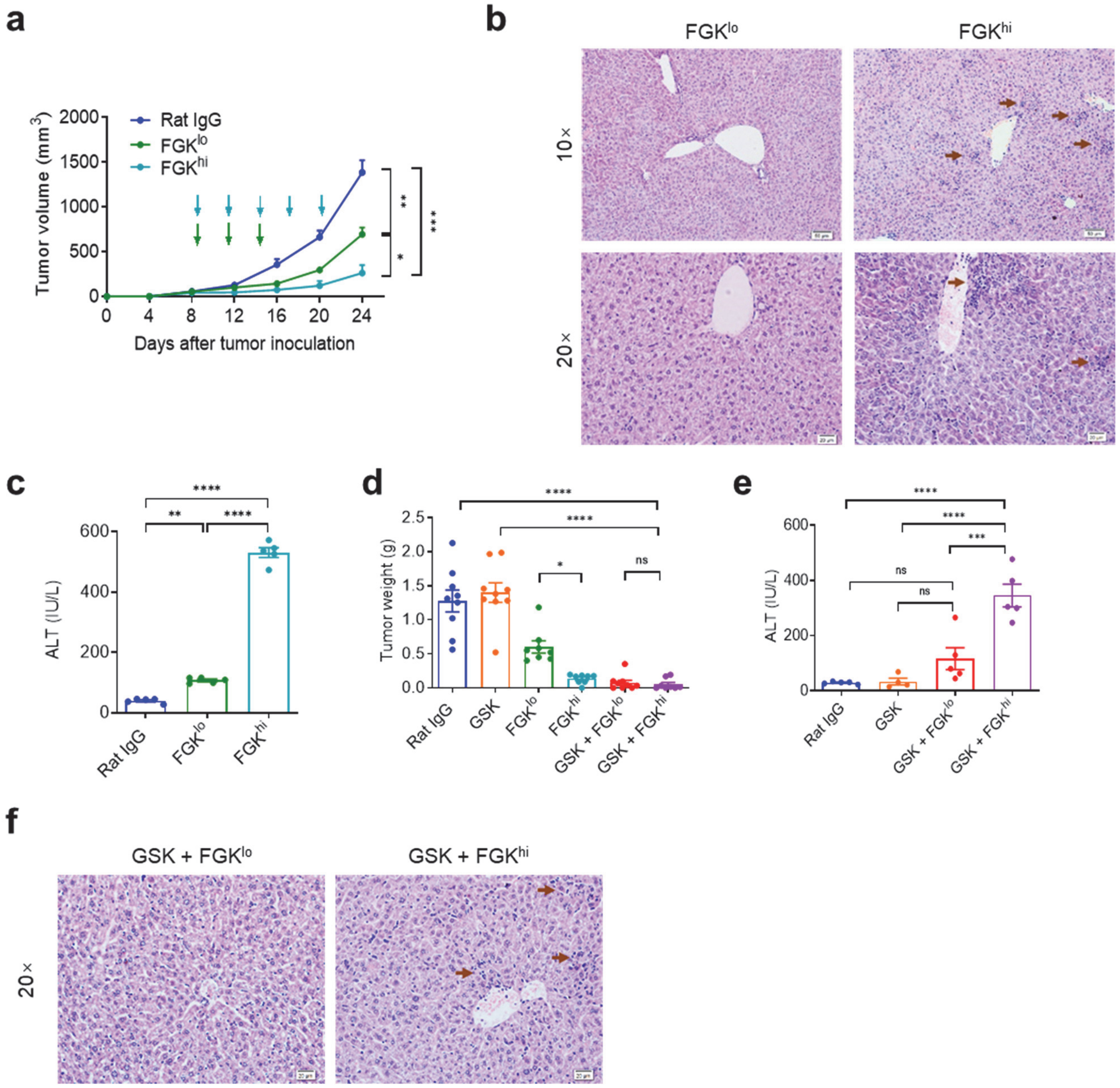

Supplementary Fig. S1. Agonistic anti-CD40 mAb induces improved antitumor effects without increasing toxicity when used in combination with PPARδ, but not PPARγ, inhibitor.

(a-c) C57BL/6 mice were inoculated s.c. with 7×10^5 B16 cells, followed 8 days later by treatment with peritumoral injection (s.c.) of FGK at an accumulated dose of 75 µg (25 µg per day at day 8, 11 and 14; FGK^{lo}) or 200 µg (40 µg per day at day 8, 11, 14, 17 and 20; FGK^{hi}), or Rat IgG at an accumulated dose of 200 µg (40 µg per day at day 8, 11, 14, 17 and 20) (n=5). (a) Tumor growth. (b) Liver histology (H&E; The arrows point to the tissue damage area with inflammatory cells infiltration) and (c) serum ALT levels examined at 3 days after the last injection of FGK. (d-f) C57BL/6 mice (n=8-9) were inoculated B16 cells and treated as indicated in Fig 1b. (d) Tumor weight at the end of experiment (0 indicates no tumor found). (e) Serum ALT levels and (f) liver histology (H&E) examined at 24 hours after the last injection of GSK. Data presented are mean ± SEM or representative photographs. *P* values were determined by two-way ANOVA with Tukey's multiple comparisons test

(a) or one-way ANOVA with Tukey's multiple comparisons test (c-e). Shown are data from a representative of two (a-c) or three (d-f) independent experiments.

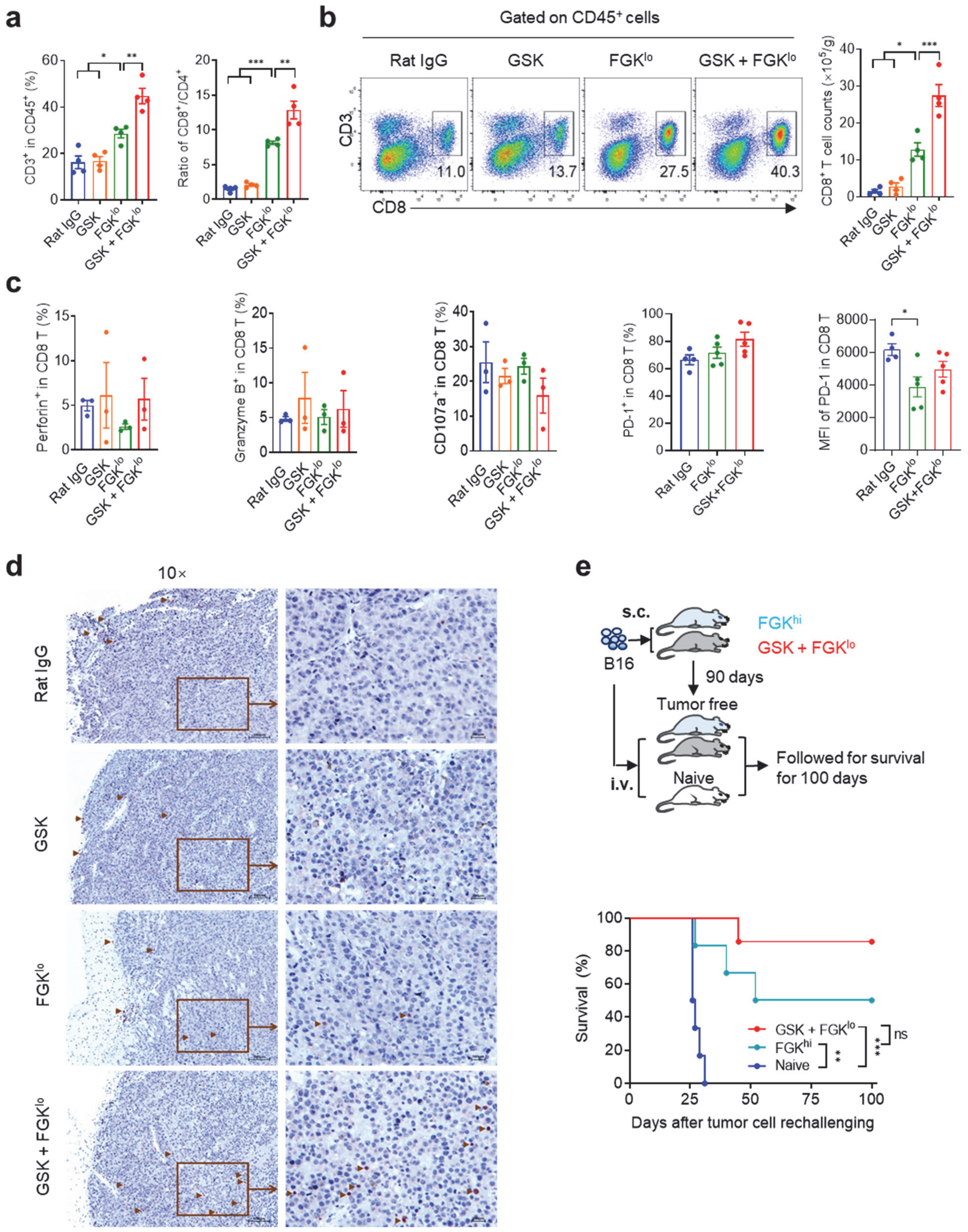

Supplementary Fig. S2. Combination treatment with immunotherapy and PPARδ inhibitor promotes tumor infiltration of CD8$^+$ T cells and anti-tumor immune memory responses.

(a-d) C57BL/6 mice (n=8-9) were inoculated B16 cells and treated as indicated in Fig 1b. Tumor was

dissected at day 24 and analyzed by flow cytometry and IHC. (a) Frequencies (%) of tumor-infiltrating $CD3^+$ T cells and ratios of $CD8^+/CD4^+$ T cells. (b) Representative staining profiles (left) and numbers (per gram tumor tissue) of tumor-infiltrating $CD8^+$ T cells (right). (c) Expression of perforin, granzyme B, CD107a and PD1 in tumor-infiltrating $CD8^+$ T cells. (d) IHC images showing intratumoral distribution of $CD8^+$ TILs (arrows point to T cells). (e) Tumor-free mice were obtained from multiple experiments, in which mice were initially inoculated (s.c.) with B16 melanoma and treated with low-dose FGK plus GSK (n=7) or with high-dose FGK alone (n=6); the overall proportions of tumor-free mice were 32% for the former group and 29% for the latter. These tumor-free mice were rechallenged with i.v. injection of 7×10^5 B16 cells 90 days after initial tumor inoculation, and age matched naïve mice (n=6) were used as the controls. Shown are schematic diagram of experiment setup (top) and Kaplan-Meier curves generated for survival data (bottom). Data are mean ± SEM or representative staining profiles or images. *P* values were determined by one-way ANOVA with Tukey's multiple comparisons test (a-c) or the log-rank test (e). Shown are data from a representative of two (e) or three independent experiments (a-d).

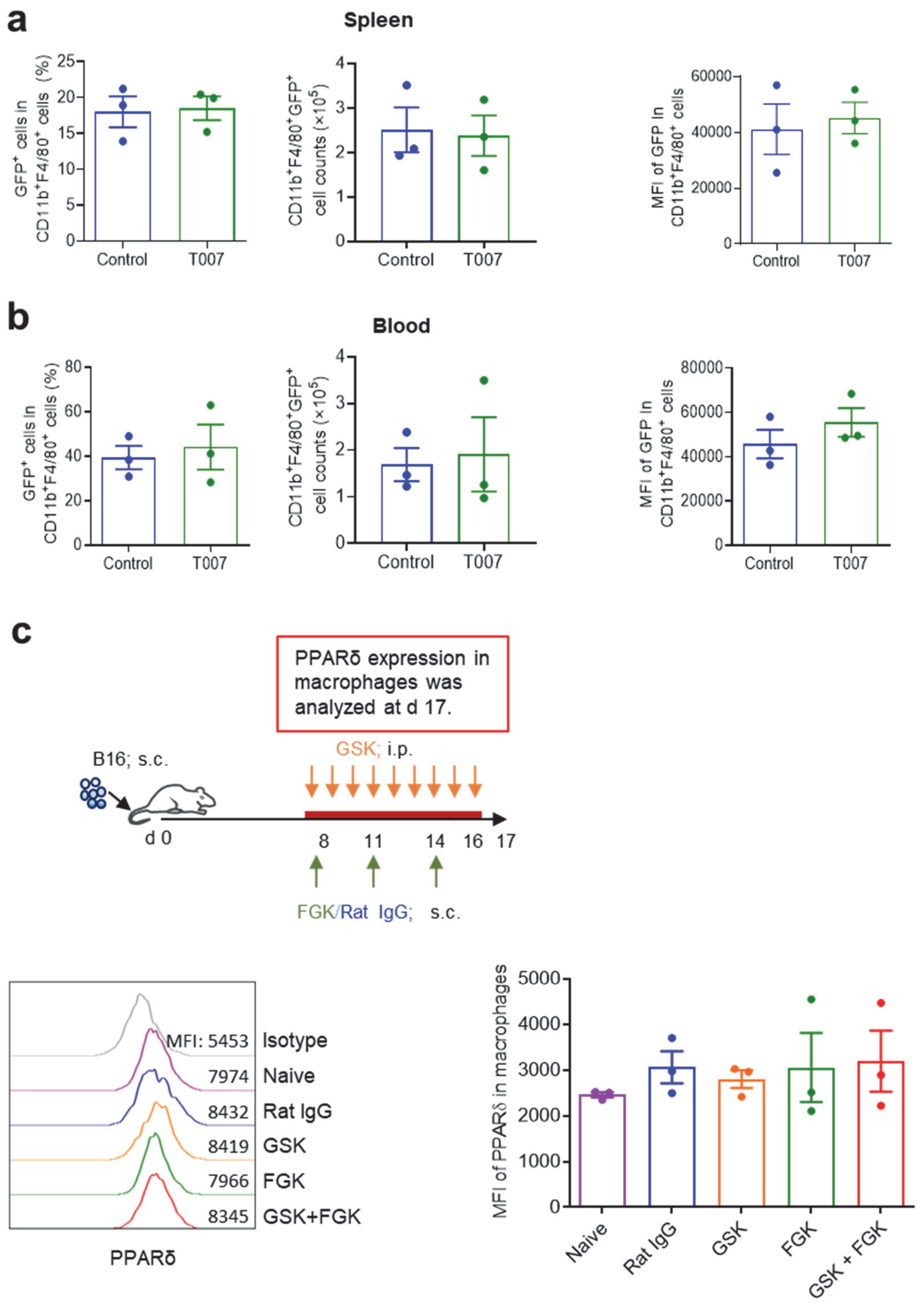

Supplementary Fig. S3. PPARγ and PPARδ expression in macrophages of tumor-bearing mice is not affected by FGK or GSK.

(a-b) PPARγ-GFP reporter mice (n=3) were inoculated with 7×10^5 B16 cells, untreated (control) or treated with T007 (45 nmol; daily for 1 week), and analyzed 1 day after last T007 injection for frequencies, quantifications (left) and levels (MFI; right) of GFP expression in $CD11b^+F4/80^+$ cells from spleens (a) and PBMCs (b). (c) Schematic diagram of experiment setup (top). Levels (MFI) of

PPARδ expression in dLN CD11b$^+$F4/80$^+$ macrophages of naïve and tumor-bearing mice (at day 17) (bottom, n=3). All data are mean ± SEM. *P* values were determined by unpaired two-tailed Student's t test (a and b) or one-way ANOVA with Tukey's multiple comparisons test (c). Shown are data from a representative of two (a, b) or three (c) independent experiments.

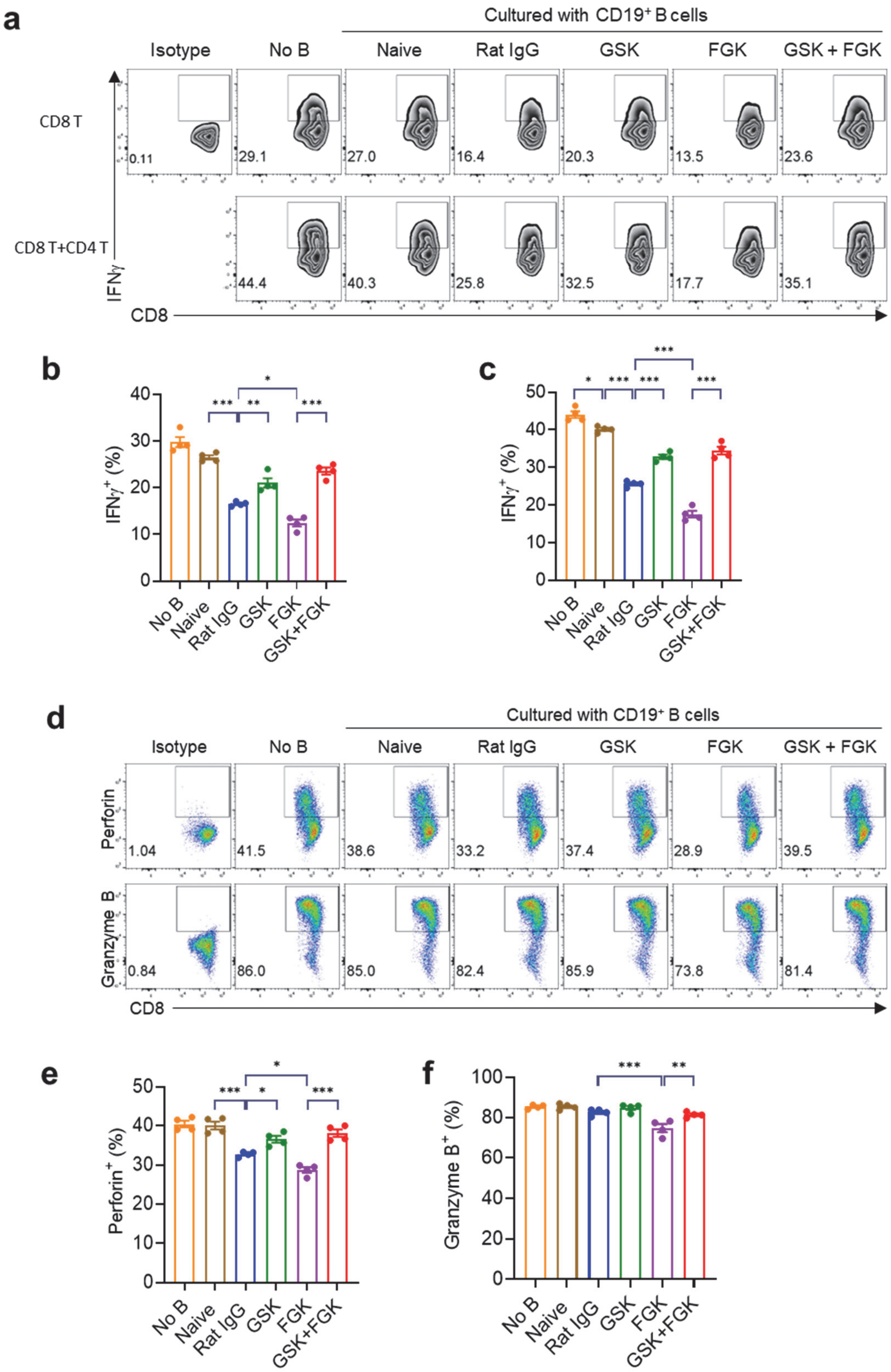

Supplementary Fig. S4. GSK attenuates the ability of tumor-associated B cells to inhibit CD8 T cell function.

C57BL/6 mice (n=8) were inoculated B16 cells and treated as indicated in Fig. 1b. dLNs (inguinal

LNs) were harvested at day 17 and assessed for their effects on CD8 T cells. (a-c) $CD19^+$ B cells were sorted from pooled dLNs of the indicated groups and cultured with CD8 T cells or with $CD4^+CD25^-$ and $CD8^+$ T cells in the presence of anti-CD3/CD28 dynabeads for 3 days, then CD8 T cells were analyzed by flow cytometry for IFNγ production. Shown are representative FACS profiles (a) and percentages of $IFN\gamma^+$ cells in $CD8^+$ T cells that were cultured alone (b) or with $CD4^+CD25^-$ T cells (c). (d-f) $CD19^+$ B cells were sorted from pooled dLNs of the indicated groups and examined for the ability to inhibit perforin and granzyme B production of $CD8^+$ T cells in response to CD3/CD28 stimulation. Shown are representative FACS profiles (d) and percentages of $perforin^+$ (e) and granzyme B^+ (f) cells in $CD8^+$ T cells. All data are mean ± SEM, and *P* values were determined by one-way ANOVA with Tukey's multiple comparisons test. Shown are data from a representative of two independent experiments.

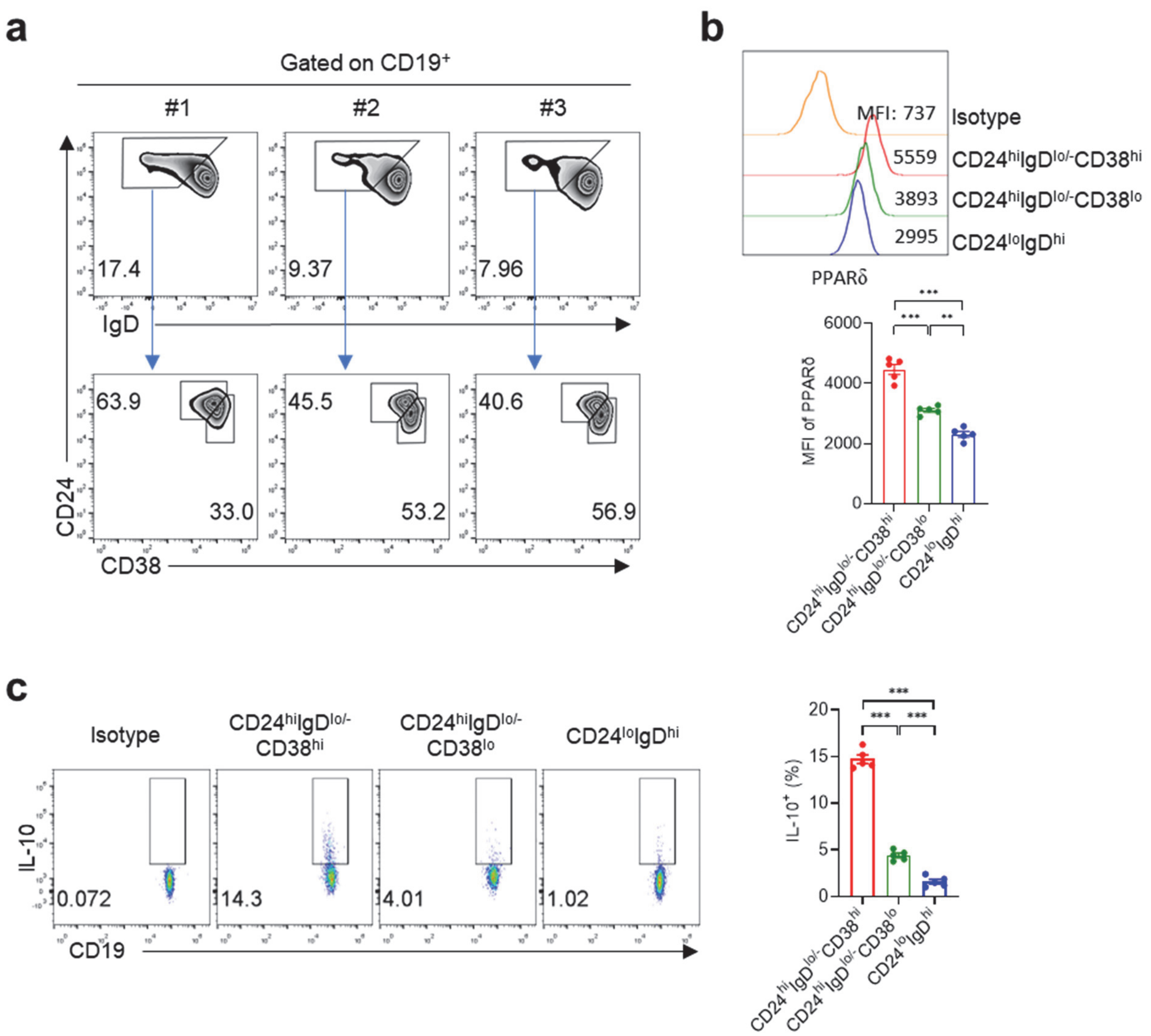

Supplementary Fig. S5. CD24^{hi}IgD$^{lo/-}$CD38^{lo} and CD24^{hi}IgD$^{lo/-}$CD38^{hi} Bregs in mouse peripheral blood.

(a) Representative profiles showing CD24 and IgD expression on gated CD19^{+} B cells (top) and CD24 and CD38 expression on gated CD19^{+}CD24^{hi}IgD$^{lo/-}$ Bregs (bottom) in naïve mouse peripheral blood. (b) Representative FACS profiles (top) and levels (MFI; bottom) of PPARδ expression (n=5). (c) Representative profiles showing IL-10 expression (left) and percentages of IL-10^{+} cells (right, n=5). All data are mean ± SEM, and *P* values were determined by one-way ANOVA with Tukey's multiple comparisons test. Shown are data from a representative of two independent experiments.

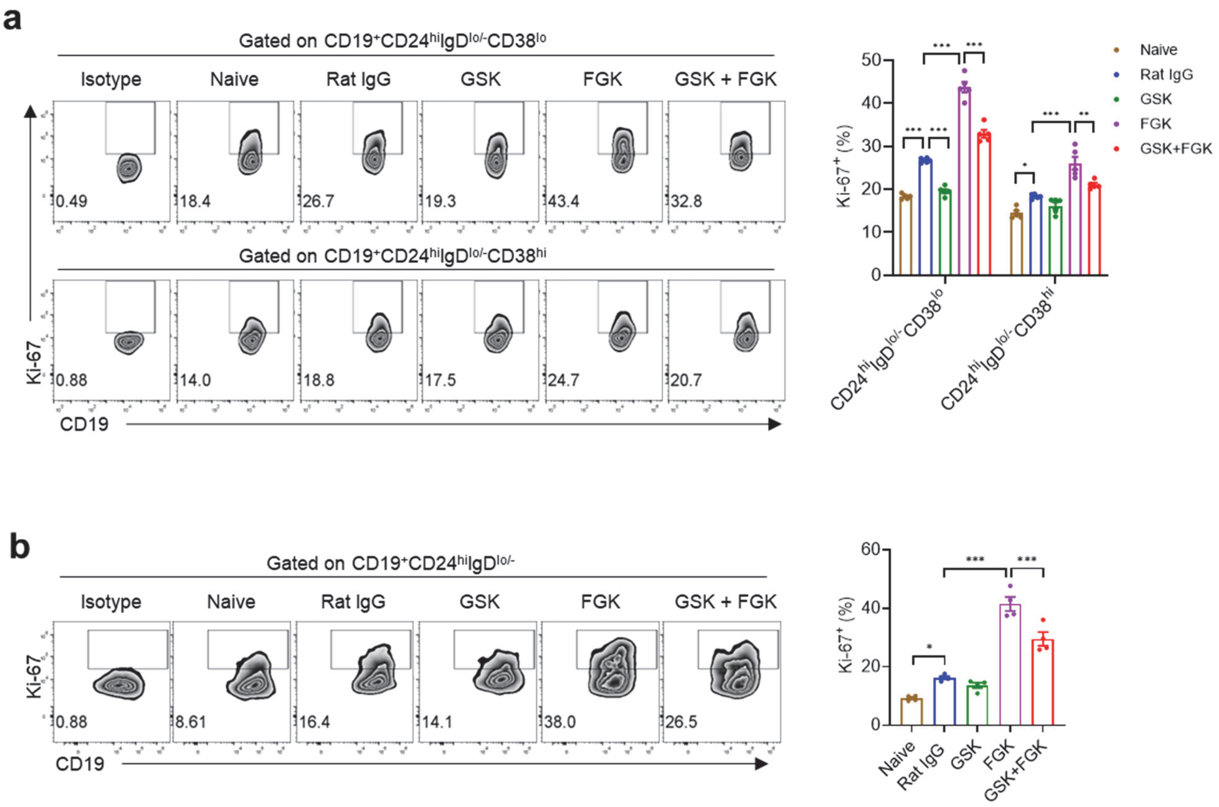

Supplementary Fig. S6. GSK inhibits Breg proliferation.

Representative Ki-67 staining profiles of gated CD19^{+}CD24^{hi}IgD$^{lo/-}$CD38^{lo} and CD19^{+}CD24^{hi}IgD$^{lo/-}$CD38^{hi} B cells (left panels) and percentages (n=4-5) of Ki-67^{+} cells (right panels) in splenic (a) and dLN (b) Breg subsets from naive or tumor-bearing mice receiving the indicated treatments. All data are mean ± SEM, and *P* values were determined by one-way ANOVA with Tukey's multiple comparisons test. Shown are data from a representative of three independent experiments.

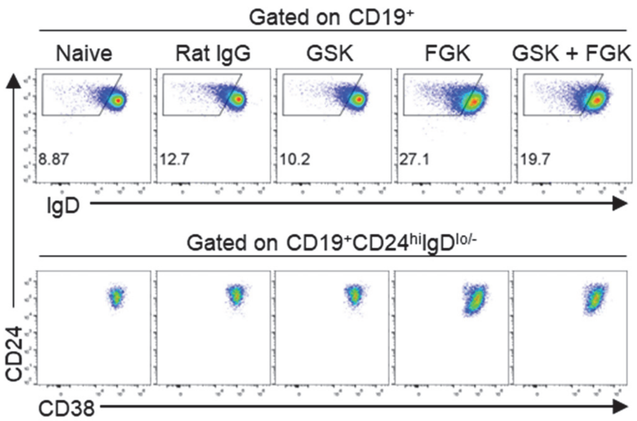

Supplementary Fig. S7. dLNs from tumor-bearing mice lack CD24^{hi}IgD$^{lo/-}$CD38^{hi} Bregs. Representative profiles showing CD24 and IgD expression on gated CD19^{+} B cells (top, these results are also presented in Fig. 3f) and CD24 and CD38 expression on gated CD19^{+}CD24^{hi}IgD$^{lo/-}$ Bregs (bottom) in dLN cells. Shown are data from a representative of two independent experiments.

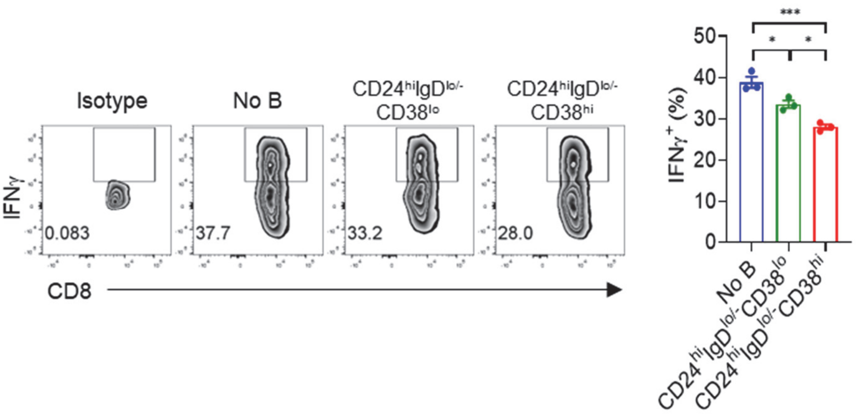

Supplementary Fig. S8. CD24^{hi}IgD$^{lo/-}$CD38^{lo} and CD24^{hi}IgD$^{lo/-}$CD38^{hi} Bregs inhibit CD8^{+} T cell activation.

CD19^{+}CD24^{hi}IgD$^{lo/-}$CD38^{lo} and CD19^{+}CD24^{hi}IgD$^{lo/-}$CD38^{hi} B cells were sorted from spleen cells of naïve WT mice, and examined for the ability to inhibit T cell activation by measuring IFNγ production of CD8^{+} T cells in response to CD3/CD28 stimulation. Shown are representative FACS profiles (left) and percentages (mean ± SEM; right) of IFNγ^{+} cells in CD8^{+} T cells. *P* values were determined by one-way ANOVA with Tukey's multiple comparisons test. Shown are data from a representative of two independent experiments.

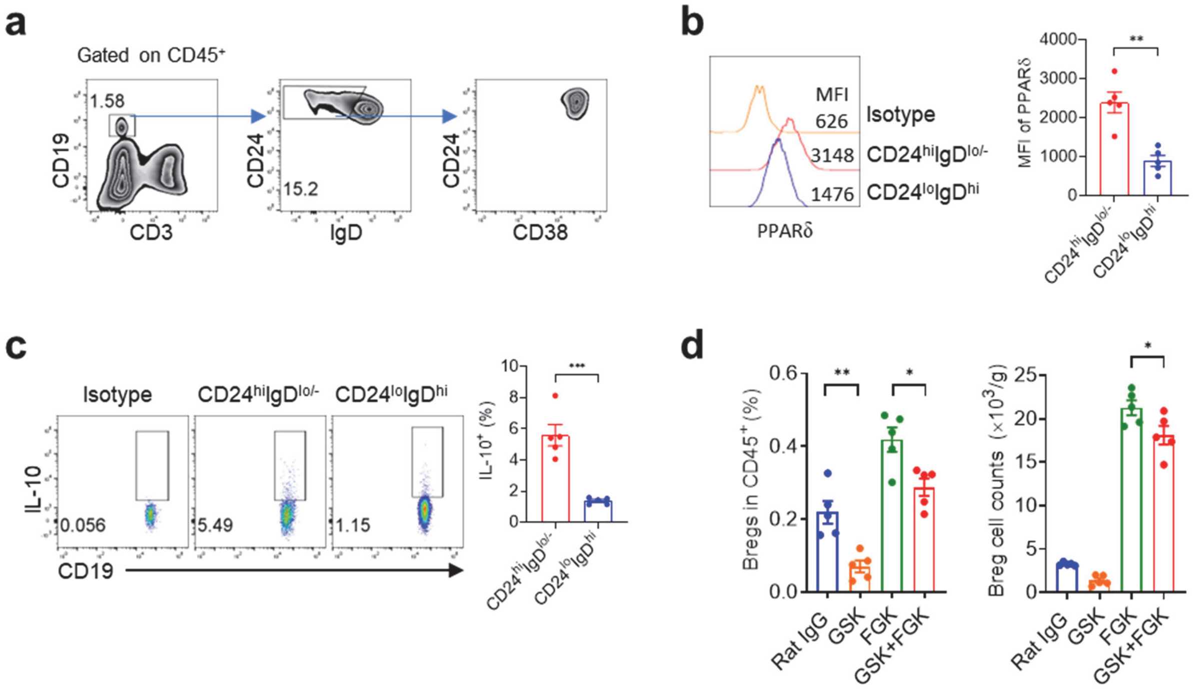

Supplementary Fig. S9. Characterization of tumor-infiltrating Bregs.

(a-d) C57BL/6 mice (n=5) were inoculated B16 cells and treated as indicated in Fig 1b. Tumor was dissected at day 17 and analyzed by flow cytometry. (a) Representative profiles showing the percentage of $CD19^+$ B cells on gated $CD45^+$ cells (left), CD24 and IgD expression on gated $CD19^+$ B cells (middle) and CD24 and CD38 expression on gated $CD19^+CD24^{hi}IgD^{lo/-}$ Bregs (right) in the tumor from a representative mouse treated with Rat IgG. (b) Representative FACS profiles (left) and levels (MFI; right; n=5) of PPARδ expression in indicated B cell subsets in the tumors from mice treated with Rat IgG. (c) Representative FACS profiles showing IL-10 expression (left) and percentages of $IL\text{-}10^+$ cells (right; n=5) in indicated tumor-infiltrating B cell subsets from mice treated with Rat IgG. (d) Frequencies (left) and numbers (per gram tumor tissue; right) of tumor-infiltrating Bregs. All data are mean ± SEM, *P* values were determined by unpaired two-tailed Student's t test (b and c) or one-way ANOVA with Tukey's multiple comparisons test (d). Shown are data from a representative of two independent experiments.

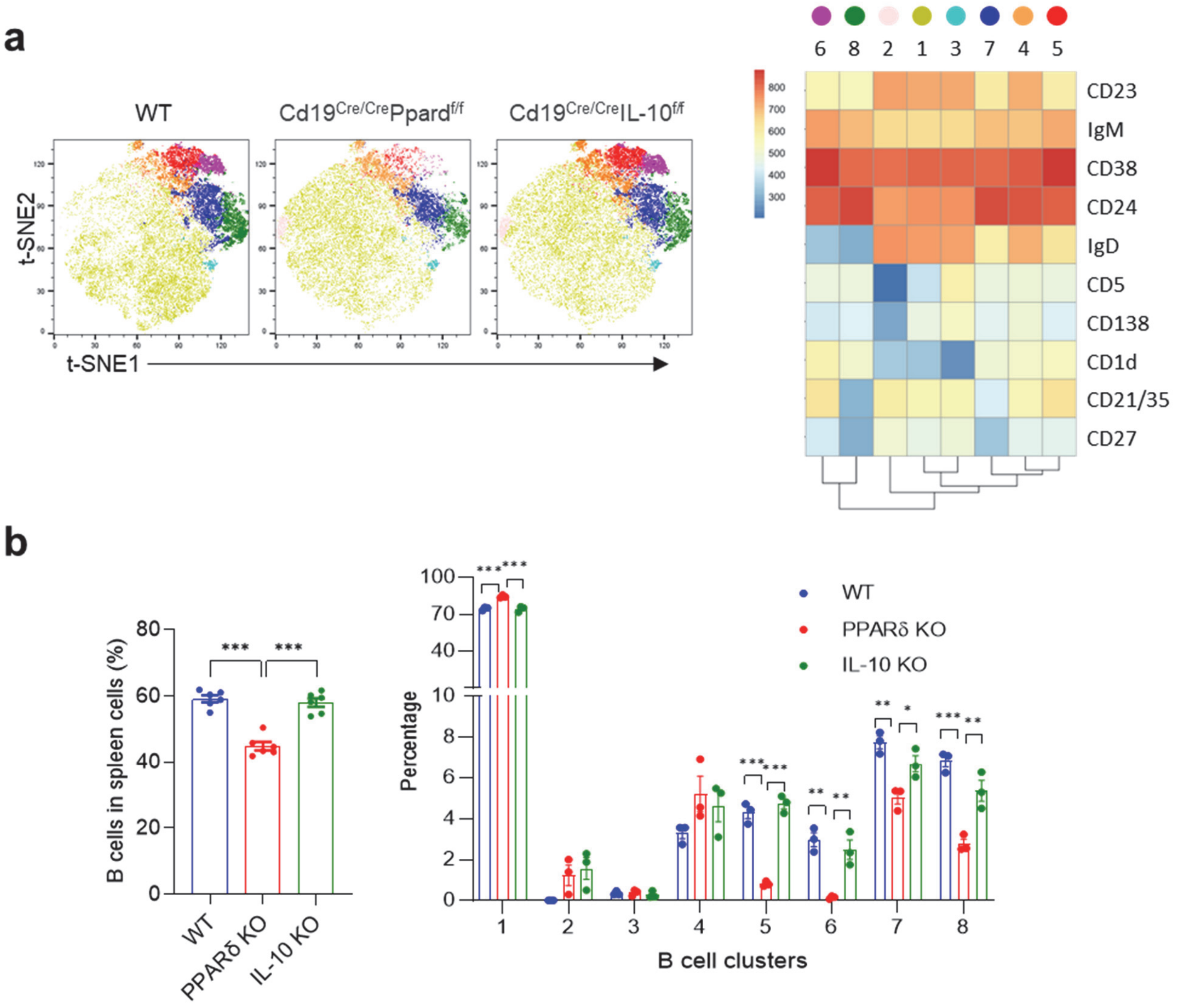

Supplementary Fig. S10. *t*-SNE analysis and clustering of splenic B cells from WT, *Cd19*$^{Cre/Cre}$*Ppard*$^{f/f}$ and *Cd19*$^{Cre/Cre}$*IL-10*$^{f/f}$ mice.

Splenic B cells from WT, *Cd19*$^{Cre/Cre}$*Ppard*$^{f/f}$ (PPARδ KO) and *Cd19*$^{Cre/Cre}$*IL-10*$^{f/f}$ (IL-10 KO) mice were stained with various antibodies, and then analyzed by flow cytometry. (a) Representative t-SNE plots of the identified B cell clusters based on the expression of CD23, IgM, CD38, CD24, IgD, CD5, CD138, CD1d, CD21 and CD27 (left), and heat map showing relative expression levels of the indicated markers by the indicated $CD19^+$ B cell clusters (right). (b) Percentages of $CD19^+$ B cells in spleens (left) and of B cell clusters in splenic $CD19^+$ B cells (right). All data are mean ± SEM, and *P* values were determined by one-way ANOVA with Tukey's multiple comparisons test. Shown are data from a representative of three independent experiments.

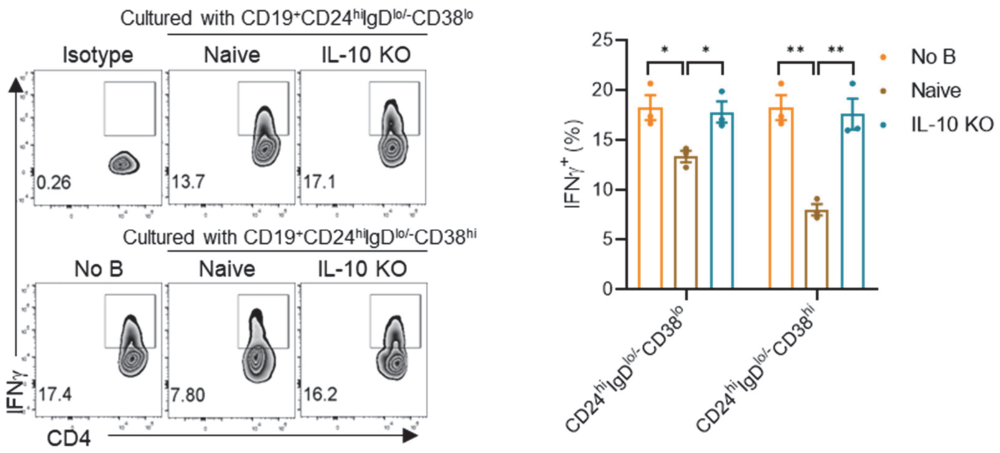

Supplementary Fig. S11. Bregs from *Cd19*$^{Cre/Cre}$*IL-10*$^{f/f}$ mice fail to inhibit T cell activation. $CD19^+CD24^{hi}IgD^{lo/-}CD38^{lo}$ and $CD19^+CD24^{hi}IgD^{lo/-}CD38^{hi}$ B cells were sorted from naïve WT and *Cd19*$^{Cre/Cre}$*IL-10*$^{f/f}$ (IL-10 KO) mice, and examined for the ability to inhibit T cell activation by measuring IFNγ production of $CD4^+CD25^-$ T cells in response to CD3/CD28 stimulation. Shown are representative FACS profiles (left) and percentages (right) of $IFN\gamma^+$ cells in $CD4^+$ T cells. All data are mean ± SEM, and *P* values were determined by one-way ANOVA with Tukey's multiple comparisons test. Shown are data from a representative of two independent experiments.

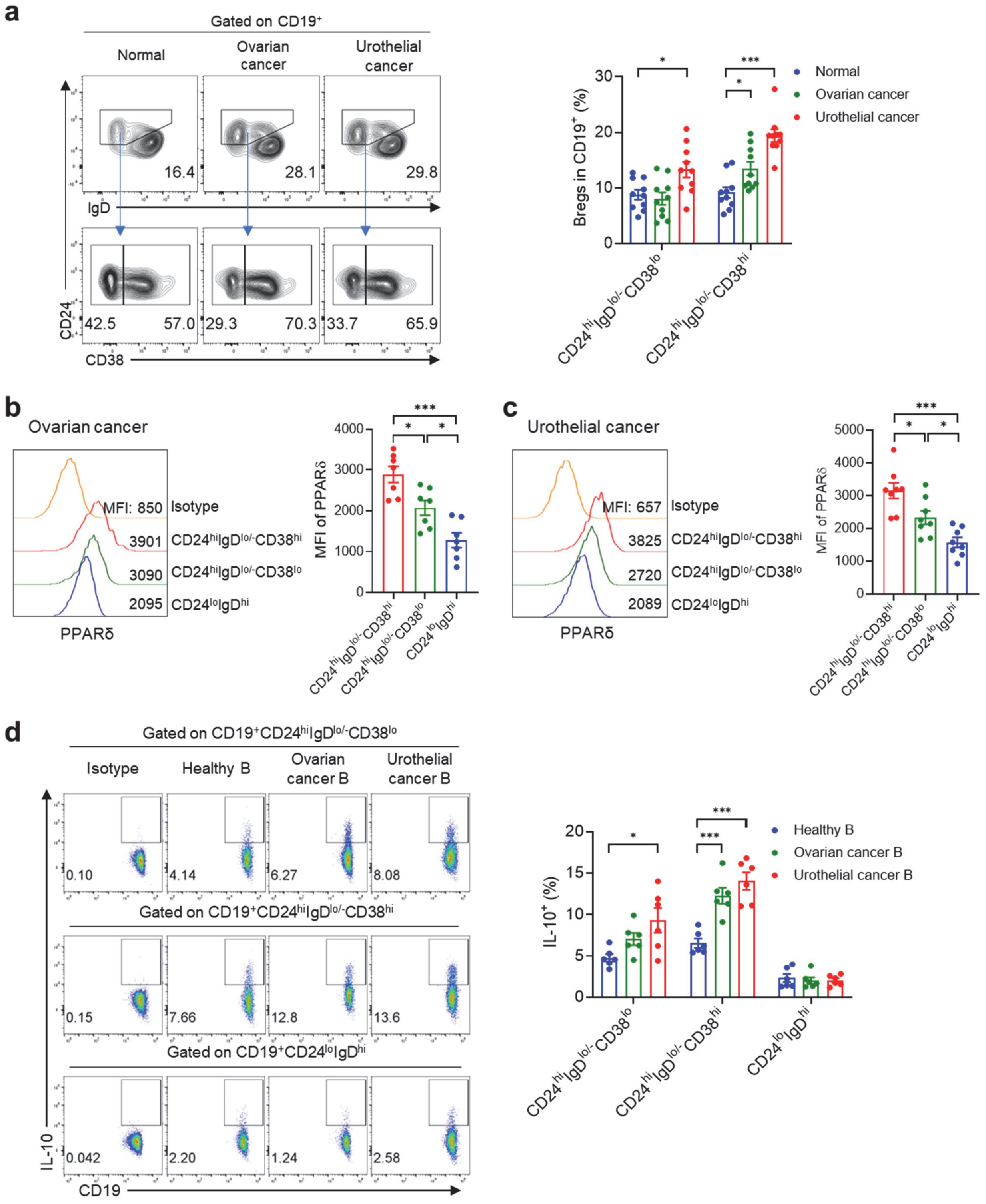

Supplementary Fig. S12. Bregs from patients with ovarian or urothelial cancer express higher PPARδ and IL-10 than $CD19^+CD24^{lo}IgD^{hi}$ B cells.

(a) Representative profiles showing CD24, IgD and CD38 expression on gated $CD19^+$ B cells (left) and percentages of $CD19^+CD24^{hi}IgD^{lo/-}CD38^{lo}$ and $CD19^+CD24^{hi}IgD^{lo/-}CD38^{hi}$ Bregs (right) in

peripheral blood of healthy subjects, ovarian cancer and urothelial cancer patients (n=10 per group). (b-c) Representative FACS profiles (left) and levels (MFI; right) of PPARδ expression in gated $CD19^{+}CD24^{hi}IgD^{lo/-}CD38^{lo}$, $CD19^{+}CD24^{hi}IgD^{lo/-}CD38^{hi}$ and $CD19^{+}CD24^{lo}IgD^{hi}$ B cells from peripheral blood of ovarian cancer (b, n=7) and urothelial cancer patients (c, n=8). Shown are combined data from two independent experiments. (d) Representative profiles showing IL-10 staining (left) and percentages of IL-10^{+} cells (right) in the indicated B cell populations (n=6 per group). Shown are data from a representative of two independent experiments. All data are mean ± SEM, and *P* values were determined by one-way ANOVA with Tukey's multiple comparisons test.

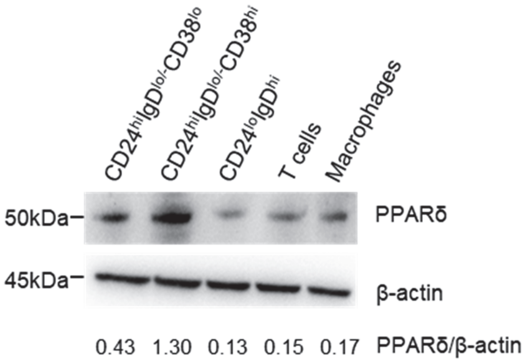

Supplementary Fig. S13. PPARδ expression in IL-10$^+$ Bregs is markedly greater than in other lymphocytes and macrophages.

The indicated B cell subsets, CD3$^+$ T cells and F4/80$^+$ macrophages were sorted from naïve mouse spleens and measured for PPARδ expression by western blot. Shown are representative results of two independent experiments.

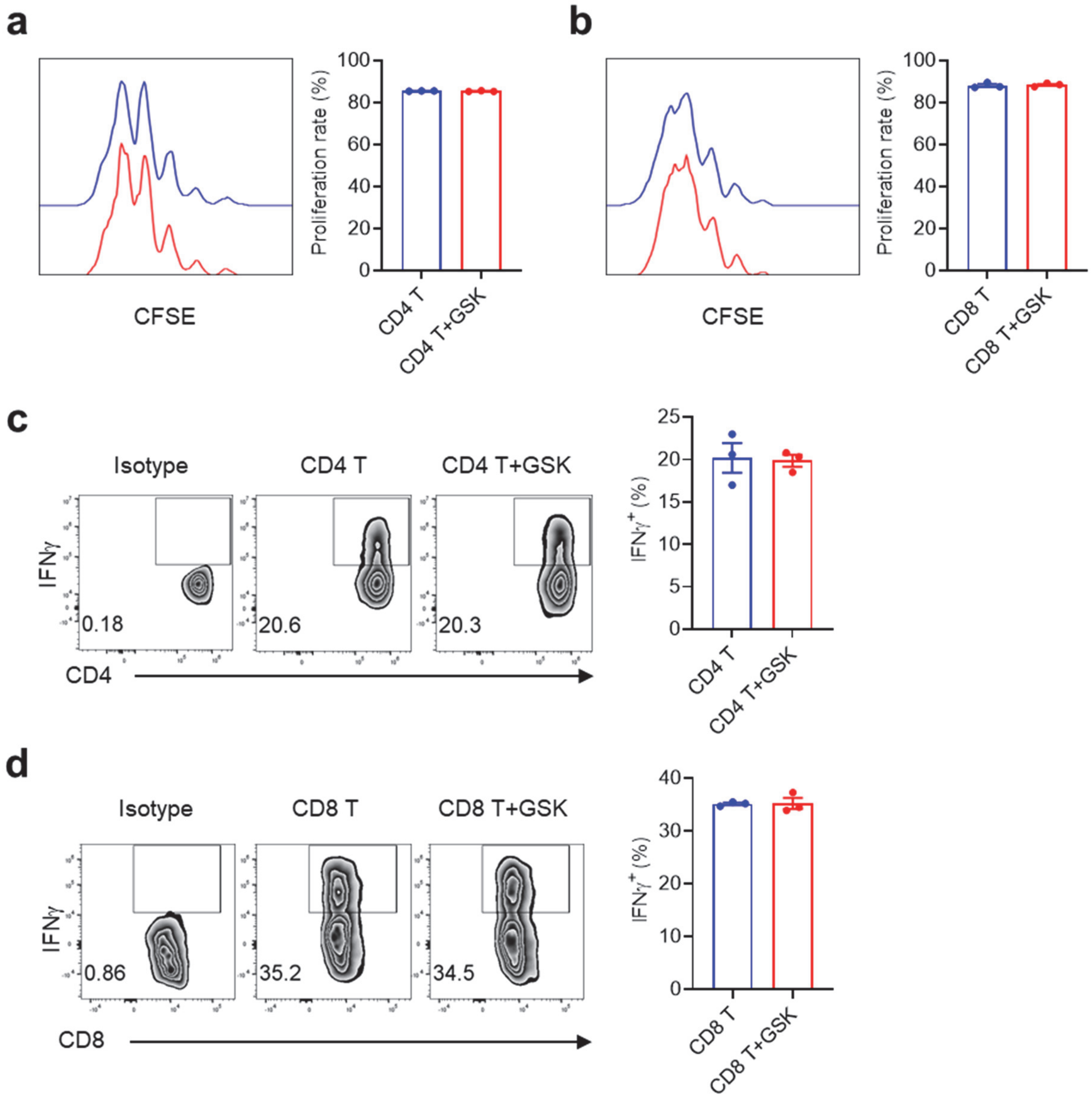

Supplementary Fig. S14. GSK didn't inhibit function of activated T cells.

$CD4^+CD25^-$ T or $CD8^+$ T cells were sorted from naïve mouse spleens and labeled with CFSE, then stimulated with anti-CD3/CD28 mAbs in the presence or absence of GSK for 3 days. (a, b) Shown are representative FACS profiles (left panels) and cell proliferation rates (right panels) of $CD4^+$ (a) and $CD8^+$ (b) T cells. (c, d) Shown are representative FACS profiles (left panels) and percentages (right panels) of $IFN\gamma^+$ cells in $CD4^+$ (c) or $CD8^+$ (d) T cells. All data are mean ± SEM (n=3), and *P* values were determined by unpaired two-tailed Student's t test. Shown are data from a representative of three independent experiments.

Supplementary Table S1. Characteristics for patients with non-small-cell lung cancer

No.	Gender	Age	Subtype	Stage	Status
1	Male	56	Adenocarcinoma	IV	Treatment naive
2	Male	63	Squamous cell carcinoma	IIIB	Treatment naive
3	Male	70	Squamous cell carcinoma	IV	Treatment naive
4	Male	64	Adenocarcinoma	IV	Treatment naive
5	Male	55	Adenocarcinoma	IIIB	Treatment naive
6	Male	58	Adenocarcinoma	IV	Treatment naive
7	Male	55	Adenocarcinoma	IIIB	Treatment naive
8	Female	63	Squamous cell carcinoma	IV	Treatment naive
9	Male	41	Adenocarcinoma	IV	Treatment naive
10	Male	66	Squamous cell carcinoma	IV	Treatment naive
11	Male	42	Adenosquamous carcinoma	IVB	Treatment naive
12	Male	55	Adenocarcinoma	IVB	Disease progression untreated
13	Male	69	Poorly differentiated carcinoma	IVB	Treatment naive
14	Male	57	Adenocarcinoma	IIIB	Disease progression untreated
15	Male	68	Squamous cell carcinoma	IIB	Treatment naive
16	Male	57	Adenocarcinoma	IIIB	Disease progression untreated
17	Male	51	Adenocarcinoma	IVB	Treatment naive
18	Male	60	Adenocarcinoma	IVA	Treatment naive
19	Male	71	Squamous cell carcinoma	IIIB	Treatment naive
20	Male	51	Squamous cell carcinoma	IIIB	Treatment naive
21	Female	51	Adenocarcinoma	IVB	Disease progression untreated
22	Male	71	Adenocarcinoma	IVB	Treatment naive
23	Female	54	Adenosquamous carcinoma	IIIB	Treatment naive
24	Male	57	Squamous cell carcinoma	IIIA	Disease progression untreated
25	Male	66	Adenocarcinoma	IVB	Disease progression untreated
26	Male	73	Adenocarcinoma	IVA	Treatment naive
27	Male	76	Adenocarcinoma	IVA	Treatment naive
28	Male	76	Adenosquamous carcinoma	IVB	Treatment naive
29	Male	56	Sarcomatoid carcinoma	IVB	Treatment naive
30	Male	58	Squamous cell carcinoma	IIIB	Treatment naive
31	Male	67	Squamous cell carcinoma	IIIA	Treatment naive
32	Male	56	Squamous cell carcinoma	IIIB	Treatment naive
33	Male	56	Squamous cell carcinoma	IVA	Treatment naive

34	Female	71	Adenocarcinoma	IVA	Disease progression untreated
35	Male	61	Sarcomatoid carcinoma	IVB	Treatment naive
36	Male	62	Squamous cell carcinoma	IIIB	Disease progression untreated
37	Male	60	Adenocarcinoma	IIIB	Treatment naive
38	Female	70	Adenocarcinoma	IVA	Disease progression untreated
39	Male	63	Squamous cell carcinoma	IIIB	Treatment naive

Supplementary Table S2. Characteristics for patients with ovarian cancer

No.	Gender	Age	Subtype	Stage	Status
1	Female	48	High grade serous ovarian carcinoma	IIIC	Treatment naive
2	Female	58	High grade serous ovarian carcinoma	IVA	Disease progression untreated
3	Female	60	High grade serous ovarian carcinoma	IVB	Treatment naive
4	Female	57	High grade serous ovarian carcinoma	IIIC	Disease progression untreated
5	Female	68	High grade serous ovarian carcinoma	IIIB	Treatment naive
6	Female	57	High grade serous ovarian carcinoma	IIIC	Disease progression untreated
7	Female	51	High grade serous ovarian carcinoma	IVB	Disease progression untreated
8	Female	60	High grade serous ovarian carcinoma	IVA	Disease progression untreated
9	Female	62	High grade serous ovarian carcinoma	IIIB	Treatment naive
10	Female	55	Ovarian clear cell carcinoma	IIIB	Treatment naive

Supplementary Table S3. Characteristics for patients with urothelial cancer

No.	Gender	Age	Subtype	Stage	Status
1	Male	65	High-grade urothelial cancer	IV	Treatment naive
2	Female	62	High-grade urothelial cancer	IV	Treatment naive
3	Male	70	High-grade urothelial cancer	IV	Treatment naive
4	Male	63	High-grade urothelial cancer	IV	Treatment naive
5	Male	58	High-grade urothelial cancer	IV	Treatment naive
6	Male	58	High-grade urothelial cancer	IV	Treatment naive
7	Male	64	High-grade urothelial cancer	IV	Treatment naive
8	Female	68	High-grade urothelial cancer	IV	Treatment naive
9	Male	51	High-grade urothelial cancer	IV	Treatment naive
10	Male	66	High-grade urothelial cancer	IV	Treatment naive
